# Supplementary material for: Comparing performance of primary care clinicians in the interpretation of SPIROmetry with or without Artificial Intelligence Decision support software (SPIRO-AID): a protocol for a randomised controlled trial
Source: BMJ Open. 2024 Jul 1;14(6):e086736. doi: 10.1136/bmjopen-2024-086736 (PMC11218008; doi:10.1136/bmjopen-2024-086736)
Supplement: Supplementary data [file bmjopen-2024-086736supp001.pdf]

Supplement_Comparing performance of primary care clinicians in the interpretation of SPIROmetry with or without Artificial Intelligence Decision support software (SPIRO-AID): a protocol for a randomised controlled trial.

Supplement

Clinician letter (participant information)

Royal Brompton and Harefield

NHS Guy's and St Thomas' NHS Foundation Trust

IRAS ID: 323361

Invitation to participate in research study: A randomized controlled trial comparing diagnostic performance of primary care clinicians in the interpretation of **SPIRO**metry with or without **A**rtificial **I**ntelligence **D**ecision support software (SPIRO-AID)

Dear Clinician,

We would like to invite you to take part in our research study. This study aims to evaluate whether an artificial intelligence decision support software (ArtiQ. Spiro) improves the diagnostic accuracy of spirometry interpreted by primary care clinicians, as measured by Clinician Diagnostic Accuracy (*vs* Reference Standard). You are being invited to take part because you are a clinician working in primary care who refers patients for spirometry and/or receives spirometry results for review.

The study is being conducted by Harefield Hospital, part of Guy's and St Thomas's NHS Foundation Trust (GSTFT), in partnership with ArtiQ, a company specialising in respiratory medicine and Artificial Intelligence (AI). The Chief Investigator (CI) is Prof William Man, Professor and Honorary Consultant Respiratory Physician. The study is being funded by National Institute for Health Research (NIHR) through an AI Award in Health and Care (Phase 3- Application: Grant number AI_AWARD02204). The lead organisation for the wider research project is Guy's and St Thomas's NHS Foundation Trust. The study is also supported by the NIHR Leicester Biomedical Research Centre - Respiratory.

Please take time to read the following information carefully. Please discuss it with others, including your colleagues if you wish. Please ask us if there is anything that is not clear.

Purpose of the Study: The NHS Long Term Plan (LTP) has identified quality assured spirometry as a clinical priority through investment in training more community staff to perform and interpret spirometry more accurately. However, this process takes time and disagreement over spirometry interpretation can still occur between trained staff. The purpose of this study is to evaluate whether an AI decision support software improves the diagnostic accuracy and quality assessment of spirometry by primary care clinicians.

Taking part: If you are happy to take part in this research please click here where you will be provided with a link to an on-line platform (Qualtrics) to assess your eligibility in the study and to complete a Consent form. You will then be asked about your job role and any previous experience with spirometry, which will help the study team to randomise participants into each arm of the study. Some participants will be randomised to receive

[illegible]

Doe G, *et al. BMJ Open* 2024; 14:e086736. doi: 10.1136/bmjopen-2024-086736

Supplement_Comparing performance of primary care clinicians in the interpretation of SPIROmetry with or without Artificial Intelligence Decision support software (SPIRO-AID): a protocol for a randomised controlled trial.

50 spirometry traces for review in the usual format that they would be received. Some participants will be randomised to receive 50 spirometry traces plus an AI report (ArtiQ.Spiro). The 50 spirometry traces will be from a de-identified retrospective dataset. We anticipate that reviewing the traces will take approximately one hour of your time.

Expenses and payments: Participants will be offered reimbursement (£150) for their time. Reimbursement details are provided via the Qualtrics study platform after you have completed review of the 50 traces.

Risks and benefits of taking part: The procedure proposed for this study does not affect the usual standard of care for participants. The datasets will comprise spirometry data previously collected as part of clinical spirometry pathways in primary care. As such, this retrospective data analysis, which does not involve delivery of an intervention nor a change in patient's usual clinical care, is unlikely to produce direct risk for participants.
This study will help us to understand if the addition of AI improves diagnostic accuracy and quality assessment of spirometry, informing design of spirometry pathways for the future.

Study period: The study period will be six months. When this period is over, the results will be analysed and written up for dissemination. The results will be presented at scientific meetings and will be published in medical journals. A dissemination event may be arranged and we will invite participants to attend.

Confidentiality: All information which is collected about you during the course of the research will be kept strictly confidential. Royal Brompton and Harefield Hospitals, part of Guy's and St Thomas NHS Foundation Trust is the sponsor for this study. The sponsor will act as the data controller. This means that it is responsible for looking after your information and using it properly. A web based electronic platform (Qualtrics) will be used to collect data responses from each participant reviewing the spirometry traces.

How will we use information about you?
We will need to use information from you for this research project. This information will include your name and contact details. People will use this information to do the research or to check your records to make sure that the research is being done properly.

People who do not need to know who you are will not be able to see your name or contact details. Your data will have a code number instead. We will keep all information about you safe and secure. Database access will be strictly restricted through user-specific passwords to the authorised research team members. At the end of the study, the CI or their assigned delegate will review all the data for each participant to verify that all the data are complete and correct. At this point, all data can be formally locked for analysis.

Supplement_Comparing performance of primary care clinicians in the interpretation of SPIROmetry with or without Artificial Intelligence Decision support software (SPIRO-AID): a protocol for a randomised controlled trial.

Once we have finished the study, we will keep some of the data so we can check the results. We will write our reports in a way that no-one can work out that you took part in the study.

What are your choices about how your information is used?
You can stop being part of the study at any time, without giving a reason, but we will keep information about you that we already have.
We need to manage your records in specific ways for the research to be reliable. This means that we won't be able to let you see or change the data we hold about you.

Where can you find out more about how your information is used?
You can find out more about how we use your information

- at www.hra.nhs.uk/information-about-patients/
- by asking one of the research team
- by sending an email to ig@rbht.nhs.uk, or
- by ringing us on 0207 352 8121 ext. 2610.

Additional information on the use of patient data in research in line with the General Data Protection Regulation (GDPR) is also provided here.

If you have a concern about any aspect of this study you should ask to speak with the researchers who will do their best to answer your questions [Insert local contact number].
If you remain unhappy and wish to complain formally, you can do this. Details can be obtained from the Royal Brompton and Harefield Hospitals (RBHH) Research Office *via* Email: (Insert)
Further Information and Contact Details:
If you require any further information, please do not hesitate to contact us on the details below.

Study email: Insert

Royal Brompton and Harefield Hospital Research Team
Contact [Insert TBC]

Gillian Doe (Research Programme Manager)
Respiratory BRC, Leicester **Tel:** 0116 258 3370 **Email:** ged6@leicester.ac.uk

Yours faithfully,

[Insert signature]
Professor Will Man (CI)

Supplement_Comparing performance of primary care clinicians in the interpretation of SPIROmetry with or without Artificial Intelligence Decision support software (SPIRO-AID): a protocol for a randomised controlled trial.

Consent form on Study platform

Q15

Please initial each box if you are in agreement

	Initials
I confirm that I have read and understand the information in the Clinician invitation letter dated 07/03/2023 (Version 1.2) for the above study and have had the opportunity to consider the information, ask questions and have these answered satisfactorily.	
I understand that my participation is voluntary and that I am free to withdraw at any time, without giving any reason, without medical care or legal rights being affected.	
I understand that data collected during the study may be looked at by responsible individuals from the study Sponsor or from regulatory authorities where it is relevant to my taking part in research. I give permission to these individuals to have access this data.	
I agree with the publication of the results of this study in a medical journal (all data will be published anonymously).	
I agree to take part in the above study.	

Q16

Name of Participant

Q17

Date

${date://CurrentDate/FL}

Q21

Type your full name below in lieu of a signature

Supplement_Comparing performance of primary care clinicians in the interpretation of SPIROmetry with or without Artificial Intelligence Decision support software (SPIRO-AID): a protocol for a randomised controlled trial.

Example AI-decision support software report

ARTIQ

Session ID: 120519_1987
Analysed on: 2023-03-23 14:50:25 UTC±00:00

Session Evaluation

FEV1 (Grade A) Acceptable
FVC (Grade B) Acceptable

Best Trial Results
Best Trial: 1
FEV1: 0.7 L
FVC: 2.19 L

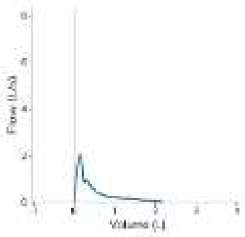

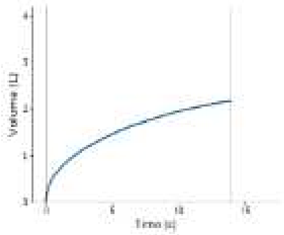

Trial Overview

	FEV1		FVC	
	Acceptable	Usable	Acceptable	Usable
Trial 1	Yes	-	Yes	-
Trial 2	Yes	-	Yes	-
Trial 3	Yes	-	No	Yes

Trial Evaluation Details

TRIAL 1
FEV1 Acceptable
FVC Acceptable

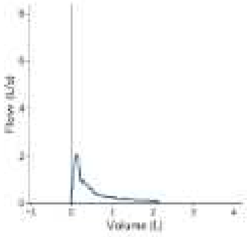

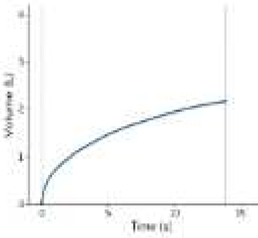

BEV Criteria: True
EOFE Criteria: True
Plateau Criteria: False
(39.35 mL change in last second)
tFE > 15s: False
FVC Repeatable: True
(30.34 mL lower than max acceptable FVC)
FIVC-FVC Criteria: None

No artefact on MEFVC

Warnings
No second inspiration

Supplement_Comparing performance of primary care clinicians in the interpretation of SPIROmetry with or without Artificial Intelligence Decision support software (SPIRO-AID): a protocol for a randomised controlled trial.

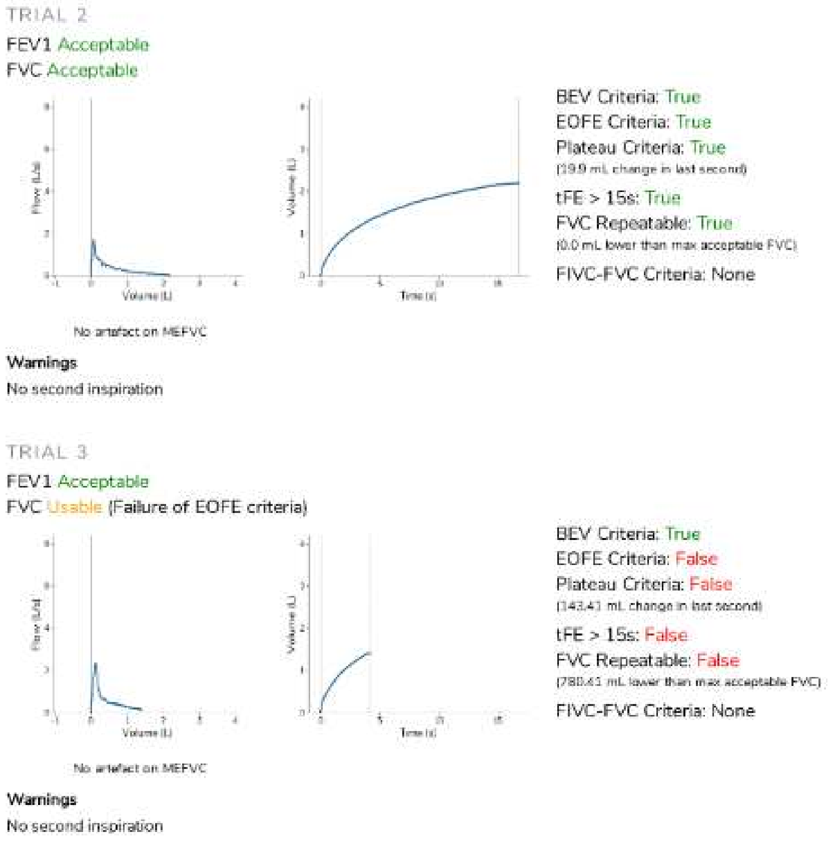

*Note no second inspiration present on these curves as this is a retrospective dataset where second inspiration was not captured

Supplement_Comparing performance of primary care clinicians in the interpretation of SPIROmetry with or without Artificial Intelligence Decision support software (SPIRO-AID): a protocol for a randomised controlled trial.

ARTIQ

Analyzed: 2023-04-18 23:33 UTC±00:00

Report ID: _120519_1987

Age: 53 **Gender:** Female **Current Smoker:** Yes **Pack-Years:** N/A

Interpretation of lung function tests

Spirometry demonstrates an obstructive pattern, but a restrictive component is not excluded.

Disease probability

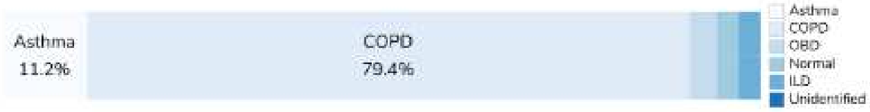

Conclusions and suggestions

Highest disease probability based on lung function: COPD

Consider referring to specialist and/or further testing as appropriate:
Careful auscultation on all lung areas for presence of rhonchi. Repeat spirometry with bronchodilator test. Perform screening laboratory (including eosinophilia). Perform X-ray of the thorax. Consider bronchodilator therapy.

Warnings

Probability of disease presence may not be accurate due to missing correct information of pack-years.

Legend

COPD	Chronic Obstructive Pulmonary Disease
OBD	Other Obstructive Diseases (including: cystic fibrosis, bronchiectasis, bronchiolitis)
Normal	Normal lung function
ILD	Interstitial lung disease (including: idiopathic pulmonary fibrosis, nonspecific interstitial pneumonitis, sarcoidosis)
Unidentified	Unidentified (including: neuromuscular disease, pulmonary vascular disease, thoracic deformity, pleural disease)

This report is approved for clinical use in the EU.
Automatically generated by ArtiQ.PFT VERSION NOT SET • Manufactured by ArtiQ NV • Leuven, Belgium
info@ArtiQ.eu
www.ArtiQ.eu
