# Supplementary material for: Comparing performance of primary care clinicians in the interpretation of SPIROmetry with or without Artificial Intelligence Decision support software (SPIRO-AID): a protocol for a randomised controlled trial
Source: BMJ Open. 2024 Jul 1;14(6):e086736. doi: 10.1136/bmjopen-2024-086736 (PMC11218008; doi:10.1136/bmjopen-2024-086736)
Supplement: Author's manuscript [file bmjopen-2024-086736.draft_revisions.pdf]

BMJ Open is committed to open peer review. As part of this commitment we make the peer review history of every article we publish publicly available.

When an article is published we post the peer reviewers' comments and the authors' responses online. We also post the versions of the paper that were used during peer review. These are the versions that the peer review comments apply to.

The versions of the paper that follow are the versions that were submitted during the peer review process. They are not the versions of record or the final published versions. They should not be cited or distributed as the published version of this manuscript.

BMJ Open is an open access journal and the full, final, typeset and author-corrected version of record of the manuscript is available on our site with no access controls, subscription charges or pay-per-view fees (http://bmjopen.bmj.com).

If you have any questions on BMJ Open's open peer review process please email info.bmjopen@bmj.com

BMJ Open

Comparing performance of primary care clinicians in the interpretation of SPIROmetry with or without Artificial Intelligence Decision support software (SPIRO-AID): a protocol for a randomised controlled trial.

Journal:	*BMJ Open*
Manuscript ID	bmjopen-2024-086736
Article Type:	Protocol
Date Submitted by the Author:	21-Mar-2024

Complete List of Authors:	Doe, Gillian; University of Leicester, NIHR Biomedical Research Centre<br>El-Emir, E; Guy's and St Thomas' NHS Foundation Trust<br>Edwards, G; Guy's and St Thomas' NHS Foundation Trust<br>Topalovic, Marko; ARTIQ<br>Evans , Rachael; University of Leicester, NIHR Biomedical Research Centre<br>Russell, Richard; King's College London, Kings Centre for Lung Health<br>Sylvester, Karl; Cambridge University Hospitals NHS Foundation Trust, Cambridge Respiratory Physiology<br>Orshoven, K; ARTIQ<br>Sunjaya, A; The George Institute for Global Health; Guy's and St Thomas' Hospitals NHS Trust<br>Scott, D; University of Southampton, Southampton Health Technology Assessments Centre<br>Prevost, A Toby; King's College London, Nightingale-Saunders Clinical Trials and Epidemiology Unit<br>Harvey, J; Guy's and St Thomas' Hospitals NHS Trust<br>Taylor, Stephanie; Queen Mary University of London, Wolfson Institute of Population Health<br>Hopkinson, Nicholas; Imperial College London, National Heart and Lung Insititute<br>Kon, S; Hillingdon Hospitals NHS Foundation Trust, Department of Respiratory Medicine; Guy's and St Thomas' NHS Foundation Trust, Department of Respiratory Medicine<br>Jarrold, I; Asthma and Lung UK<br>Spain, N; PPI partner<br>Banya, W; Guy's and St Thomas' Hospitals NHS Trust<br>Man, William; Guy's and St Thomas' Hospitals NHS Trust; King's College London, King’s Centre for Lung Health

Keywords:	RESPIRATORY MEDICINE (see Thoracic Medicine), Primary Care < Primary Health Care, Lung Diseases

SCHOLARONE™
Manuscripts

Title: Comparing performance of primary care clinicians in the interpretation of SPIROmetry with or without Artificial Intelligence Decision support software (SPIRO-AID): a protocol for a randomised controlled trial.

Authors:

Doe G[1], El-Emir E[2], Edwards G[2], Topalovic M[3], Evans RA[1], Russell R[4], [3], Sylvester KP[5], Van Orshoven K[3], Sunjaya AP[2,6], Scott DA[7], Prevost AT[8], Harvey J[2], Taylor SJC[9], Hopkinson NS[10], Kon SS[2,11], Jarrold I[12], Spain N[13], Banya W[2,14] and Man WD-C[2, 4, 10]

Institutions:

1. NIHR Biomedical Research Centre (Respiratory Theme), Department of Respiratory Science, University of Leicester, Leicester, UK
2. Harefield Respiratory Research Group, Royal Brompton & Harefield Hospitals, Guy's and St Thomas' NHS Foundation Trust, UK
3. ARTIQ, Leuven, Belgium
4. King's Centre for Lung Health, Faculty of Life Sciences and Medicine, King's College, London.
5. Royal Papworth Hospital NHS Foundation Trust, UK
6. The George Institute for Global Health, UNSW Sydney, Australia and The George Institute for Global Health, Imperial College London, UK
7. Southampton Health Technology Assessments Centre, University of Southampton, Southampton, UK; Diabetes Research Centre, University of Leicester, Leicester, UK
8. Nightingale-Saunders Clinical Trials and Epidemiology Unit, Cicely Saunders Institute of Palliative Care, Policy and Rehabilitation, Kings College London, UK
9. Wolfson Institute of Population Health, Queen Mary University of London, London, UK
10. National Heart & Lung Institute, Imperial College London, UK
11. Department of Respiratory Medicine, The Hillingdon Hospitals NHS Foundation Trust, UK
12. Asthma + Lung UK
13. Patient and public involvement representative, UK
14. Medical Statistics, Research & Development, Royal Brompton & Harefield Hospitals, Guy's and St Thomas' NHS Foundation Trust, UK

Author for correspondence: Gillian Doe, Leicester NIHR Biomedical Research Centre – Respiratory, Glenfield Hospital, Leicester LE3 9QP

Email: ged6@leicester.ac.uk ORCID ID: 0000-0003-4782-581

For peer review only - http://bmjopen.bmj.com/site/about/guidelines.xhtml

Abstract

Introduction: Spirometry is a point-of-care lung function test that helps support the diagnosis and monitoring of chronic lung disease. The quality and interpretation accuracy of spirometry is variable in primary care. This study aims to evaluate whether artificial intelligence (AI) decision support software improves the performance of primary care clinicians in the interpretation of spirometry, against reference standard (expert interpretation)

Methods and Analysis: A parallel, two-group, statistician-blinded, randomised controlled trial of primary care clinicians in the United Kingdom, who refer for, or interpret, spirometry. People with specialist training in respiratory medicine to consultant level were excluded. A minimum target of 228 primary care clinician participants will be randomised with a 1:1 allocation to assess fifty de-identified, real-world patient spirometry sessions through an on-line platform either with (Intervention group) or without (Control group) AI decision support software report. Outcomes will cover primary care clinicians' spirometry interpretation performance including measures of technical quality assessment, spirometry pattern recognition and diagnostic prediction, compared with reference standard. Clinicians' self-rated confidence in spirometry interpretation will also be evaluated. The primary outcome is the proportion of the fifty spirometry sessions where the participant's preferred diagnosis matches the reference diagnosis. Unpaired t-tests and Analysis of Covariance will be used to estimate the difference in primary outcome between intervention and control groups.

Ethics and Dissemination: This study has been reviewed and given favourable opinion by Health Research Authority Wales (Reference: 22/HRA/5023) and registered on Clinical trials.gov (Reference: NCT05933694).

Results will be submitted for publication in peer-reviewed journals, presented at relevant national and international conferences, disseminated through social media, patient and public routes and directly shared with stakeholders.

Trial registration: NCT05933694

Strengths and Limitations:

For peer review only

- This will be the first randomised controlled efficacy study with an intervention designed to improve the performance of primary care clinicians in the interpretation of spirometry.
- This will be the first randomised controlled trial to directly test the effect of an AI-powered spirometry interpretation software in primary care.
- Real-world spirometry traces completed in primary care, irrespective of technical quality, will be used.
- The trial will be advertised and recruited nationally to maximise participation and variation in participant sample.
- The trial is conducted through an online platform and designed as a randomised controlled efficacy study rather than a real-world implementation trial.

Introduction

Chronic respiratory disease has significant negative impact on quality of life and is the third leading cause of death globally, and one of the highest contributors to economic burden for healthcare systems (1). Chronic Obstructive Pulmonary Disease (COPD) alone accounted for 3.3 million deaths worldwide in 2019, increasing 14% over the previous ten years (2). Accurate and timely diagnosis of respiratory disease is key to improving access to treatment and patient outcomes, and long delays to diagnosis for individuals with chronic lung diseases are well documented (3-5).

Spirometry is a simple, point-of-care lung function procedure recommended to support the diagnosis and monitoring of COPD and asthma, the commonest long-term respiratory conditions (6, 7). In many western and low and middle income (LMIC) countries, spirometry will be performed and interpreted in non- specialist respiratory settings. However, there is inequity in spirometry provision in primary care and community settings; historical challenges with quality, skills and competence in interpretation, and funding, have been compounded by the COVID-19 pandemic (8). Spirometry performed in primary care and community settings has been shown to not meet international criteria in over 85% of cases (9). A low level of confidence in assessing spirometry quality is also common amongst general practitioners (10). Improving prevention and early diagnosis has been identified as a priority in the Global Impact of Respiratory Disease report (11). In practice, this requires improved equity, access and quality of spirometry provision.

For peer review only - http://bmjopen.bmj.com/site/about/guidelines.xhtml

Recent work has shown that Artificial Intelligence (AI)–powered decision support software can provide automated technical quality assessment and interpretation of full lung function tests (12, 13). This software can outperform pulmonologists in diagnostic prediction from lung function tests (13). Similar AI software that interprets spirometry (usually the only lung function test accessible in many healthcare settings) may be particularly helpful in primary care to support clinicians who have generalist roles and less experience in respiratory diagnosis. Qualitative work highlighted that validation and evaluation of the effectiveness of AI software is key in engaging clinicians and commissioners to have confidence in implementation in clinical practice.

The primary objective of this study is to compare the performance of primary care clinicians in the interpretation of 50 real-world spirometry records with or without AI spirometry decision support software reports.

Methods and Analysis

Trial Design and Registration

This is a parallel, two group, randomised controlled, statistician-blinded efficacy trial to evaluate whether AI spirometry decision support software can improve the performance of primary care clinicians in their interpretation of real-world spirometry. Each clinician will be provided with the same clinical dataset of 50 de-identified, real-world patient spirometry records through an online platform (Qualtrics XM), and asked to answer questions about preferred diagnosis (i.e. diagnosis they think is most likely) and differential diagnosis (i.e. second most likely diagnosis) based on spirometry and limited clinical data, the technical quality of spirometry and the spirometry pattern. Questions will also explore clinicians' confidence in their assessments.

Participants will be allocated at random to receive either spirometry records alone or spirometry records with the addition of the AI software report. The clinical spirometry records will be de-identified (name, date of birth, address, postcode, occupation, GP, medications data removed), by a member of the clinical care team.

For peer review only

Royal Brompton and Harefield Hospitals (RBHH), Guy's and St Thomas' NHS Foundation Trust (GSTFT) will act as study sponsor and the trial has been registered with clinicaltrials.gov (NCT05933694). This study has been reviewed and given favourable opinion by Health Research Authority Wales (Reference: 22/HRA/5023).

Study Population

Potential participants will be clinicians working in primary care involved in spirometry referral and interpretation.

Eligibility Criteria

Eligibility criteria will be: 1) A clinician working in primary care (defined as >50% of job plan in primary care) in the United Kingdom who refers for or performs spirometry (typically GP, practice nurse); 2) Able to access an online study platform in order to review spirometry sessions; 3) Able to provide informed consent via the study platform.

Exclusion criterion will be: 1) Clinicians who have completed specialist training in respiratory medicine and are recognised by the General Medical Council with the right to practise as a consultant in respiratory medicine in the National Health Service.

Eligibility criteria will be used as screening questions on the on-line study platform prior to participants being able to consent to participate.

Participants will be randomised 1:1 to either control (report 50 spirometry records alone) or intervention (report same 50 spirometry records with access to an AI spirometry interpretation report for each spirometry result). Randomisation will be done by the study on-line platform at the point of consent to the study by minimising according to job role (General Practitioner: Yes or No) and spirometry accreditation from the Association for Respiratory Technology and Physiology (Performance Only, Interpretation Only, Performance and Interpretation, No accreditation) and then randomising by a random number generation algorithm performed by the on-line platform.

Recruitment and Consent

Recruitment to the study will be advertised through national and local primary care networks, professional networks, the institution website and social media will be used. By advertising and recruiting nationally, the recruitment strategy aims to engage with clinicians from diverse

For peer review only

For peer review only - http://bmjopen.bmj.com/site/about/guidelines.xhtml

backgrounds in terms of the patient population they serve, age, years of clinical experience, job role and ethnicity.

Clinicians will be sent a letter outlining the research study with the option for them to express interest in participating. The letter serves as the participant information sheet (see supplementary information) and will be sent via email. Participants who express interest in taking part will be sent a link to the on-line study platform where they will be able to complete the eligibility screening questions and, if applicable, consent form online. The study will also be advertised on the Royal Brompton and Harefield Hospitals Research website with a link available to the on-line study platform. Participants will be able to withdraw without giving any reason, and the number of participants registering but not completing the study will be recorded. Randomisation will be kept concealed until the point of the allocation of the participant to a study arm.

Setting

The study will be conducted via an online platform (Qualtrics XM).

Procedure

Study participants (participating clinicians) will independently assess the same 50 spirometry records, in the same order, through a bespoke designed online platform developed using Qualtrics XM (Seattle, USA). Participants will be blinded to the reference standards. Please see Figure 1 for example. The patient spirometry records were randomly selected from a database comprising 1122 patients undergoing spirometry in primary care and community-based respiratory clinics in Hillingdon borough between 2015-2019. An online randomizer will be used to select the 50 spirometry sessions used in the trial (https://www.randomizer.org/), according to "disease category" so that of the 50 spirometry traces selected, 40% will be from patients with COPD, 20% will have Normal spirometry, and 10% for each of the four other disease categories (Asthma, ILD, Other obstructive, Other disease/unidentifiable category), to represent a likely distribution of diagnoses in a primary care setting.

For each spirometry record, the primary care clinician participant will answer the following questions on the Qualtrics platform:

For peer review only - http://bmjopen.bmj.com/site/about/guidelines.xhtml

- What is the Technical Quality of the spirometry? (a drop-down box containing the options: Acceptable (Quality grade A or B), Not acceptable). Technical quality will be defined according to the ATS/ERS 2019 Spirometry Technical Statement, provided to participants via a link embedded in the on-line platform
  - One response for Forced expiratory volume in one second (FEV_1) and one question for Forced Vital Capacity (FVC)
- How confident are you in your technical quality assessment using a visual analogue scale (0: Not confident at all to 10: Very Confident)?
- Provide a pattern interpretation based on spirometry report received. Options: Normal, Airflow Obstruction, Possible restriction or non-specific pattern, Possible mixed disorder
- How confident are you in your pattern interpretation using a visual analogue scale (0: Not confident at all to 10: Very Confident)?
- Provide a preferred diagnosis based on spirometry report received and information. (a drop-down box containing the options: Asthma, COPD, interstitial lung disease (ILD), normal lung function, other obstructive disease, and other unidentifiable disease): **Preferred Diagnosis**
- Provide a differential diagnosis based on spirometry report received and information. (a drop-down box containing the options: Asthma, COPD, interstitial lung disease (ILD), normal lung function, other obstructive disease, and other unidentifiable disease)**: Differential Diag**nosis. The previously chosen 'preferred diagnosis' is automatically removed from the list of available options.
- How confident are you in your diagnosis using a visual analogue scale (0: Not confident at all to 10: Very Confident)

For peer review only - http://bmjopen.bmj.com/site/about/guidelines.xhtml

Figure 1. Example screen shot from Qualtrics platform

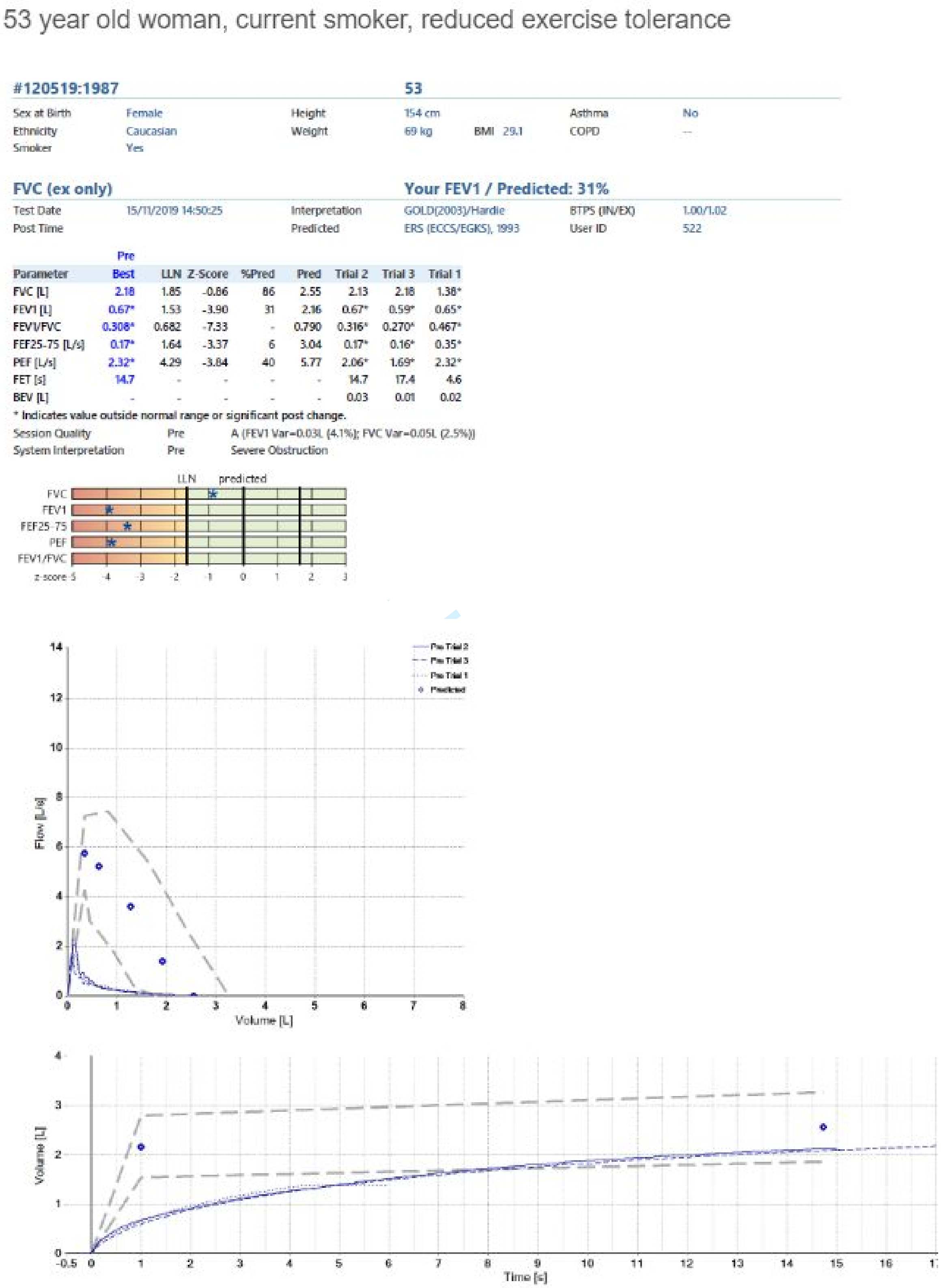

53 year old woman, current smoker, reduced exercise tolerance

#120519:1987 **53**

Sex at Birth	Female	Height	154 cm	Asthma	No
Ethnicity	Caucasian	Weight	69 kg BMI 29.1	COPD	--
Smoker	Yes				

FVC (ex only) **Your FEV1 / Predicted: 31%**

Test Date	15/11/2019 14:50:25	Interpretation	GOLD(2003)/Hardie	BTPS (IN/EX)	1.00/1.02
Post Time		Predicted	ERS (ECCS/EGKS), 1993	User ID	522

Parameter	Pre Best	LLN	Z-Score	%Pred	Pred	Trial 2	Trial 3	Trial 1
FVC [L]	2.18	1.85	-0.86	86	2.55	2.13	2.18	1.38*
FEV1 [L]	0.67*	1.53	-3.90	31	2.16	0.67*	0.59*	0.65*
FEV1/FVC	0.308*	0.682	-7.33	-	0.790	0.316*	0.270*	0.467*
FEF25-75 [L/s]	0.17*	1.64	-3.37	6	3.04	0.17*	0.16*	0.35*
PEF [L/s]	2.32*	4.29	-3.04	40	5.77	2.06*	1.69*	2.32*
FET [s]	14.7	-	-	-	-	14.7	17.4	4.6
BEV [L]	-	-	-	-	-	0.03	0.01	0.02

* Indicates value outside normal range or significant post change.

Session Quality	Pre	A (FEV1 Var=0.03L (4.1%); FVC Var=0.05L (2.5%))
System Interpretation	Pre	Severe Obstruction

What is the technical quality of FEV1?

What is the technical quality of FVC?

How confident are you in your assessment of the FEV1 and FVC technical quality using a visual analogue scale (0: Not confident at all to 10: Very Confident)?

0 1 2 3 4 5 6 7 8 9 10

Confidence

Provide a pattern interpretation based on spirometry report received.

Normal

Airflow obstruction

Possible restriction or non-specific pattern

Possible mixed disorder

How confident are you in your pattern interpretation using a visual analogue scale (0: Not confident at all to 10: Very Confident)?

0 1 2 3 4 5 6 7 8 9 10

Confidence

Provide a preferred diagnosis based on spirometry report received and information. Select one of the following:

How confident are you in your preferred diagnosis using a visual analogue scale (0: Not confident at all to 10: Very Confident)?

0 1 2 3 4 5 6 7 8 9 10

Confidence

Participants will have eight weeks to review the 50 spirometry traces; reminders will be sent at weeks three, six and seven to those who have not completed their assessments. (Figure 2).

For peer review only - http://bmjopen.bmj.com/site/about/guidelines.xhtml

Figure 2. Study schedule

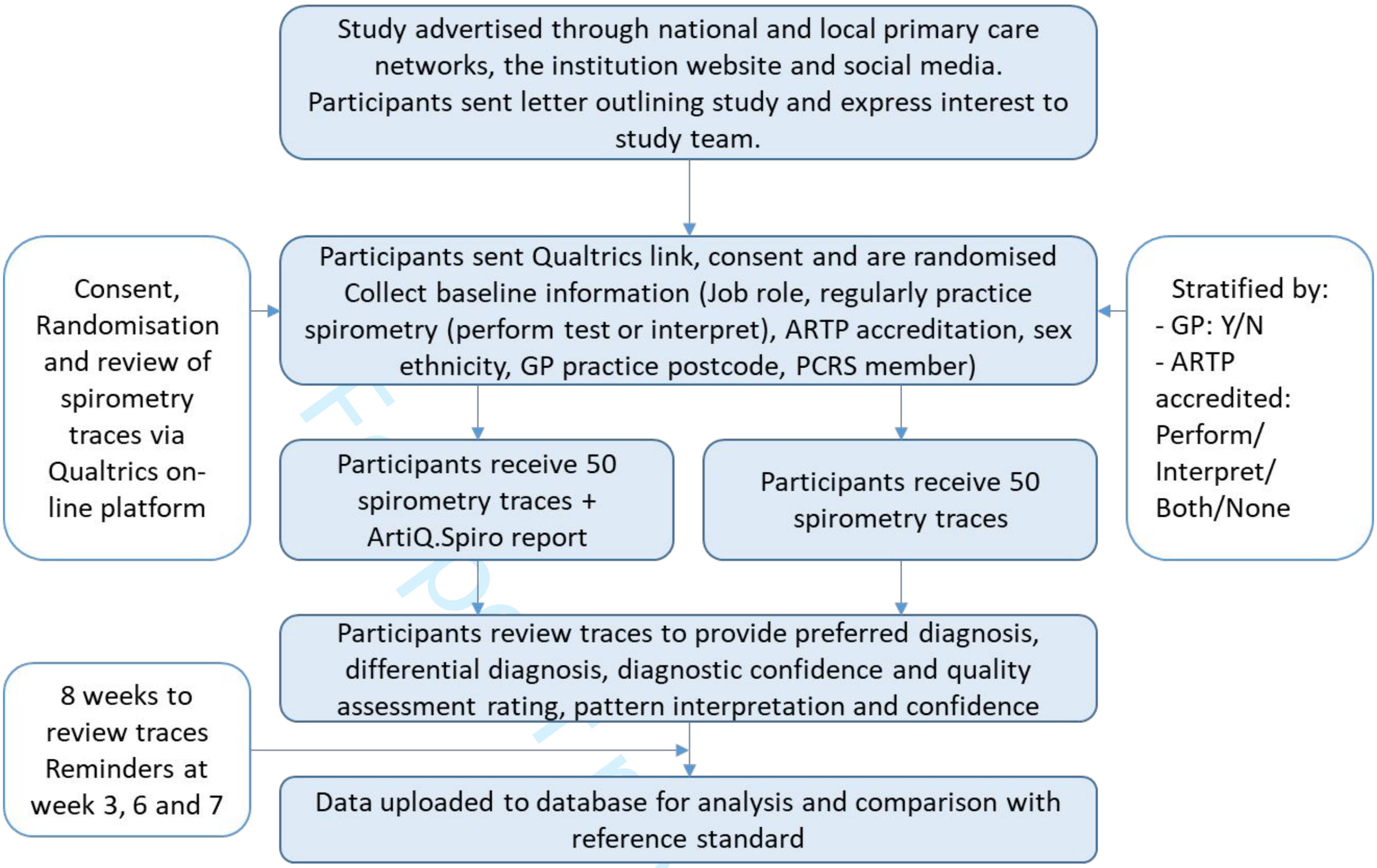

Intervention – AI spirometry software

The AI software was developed by ArtiQ (Leuven, Belgium), and provides AI-supported quality assessment and interpretation guidance of spirometry sessions (Supplement). The quality assessment component leverages deep learning methods to perform the subjective assessment of spirometry quality assessment (i.e., related to curve shape), as well as implementing the objective criteria from international guidance (i.e., related to numeric criteria). The AI component of the software mimics the subjective visual inspection of data usually performed by technicians. Per session it provides an overall session quality grade according to ATS/ERS 2019 Spirometry Technical Statement (A-F) and calculates the best trial (spirometry trace) which should be considered for diagnostic interpretation. The model was trained based on spirometry measurements from the National Health and Nutritional Examination Survey (NHANES 2011-12) (12) and validated in clinical trial settings (14).

For peer review only - http://bmjopen.bmj.com/site/about/guidelines.xhtml

The spirometry interpretation component is a decision support software focusing on the diagnostic interpretation of spirometry sessions. It provides support in pattern interpretation according to international interpretation guidelines (i.e. normal, obstructive and/or restrictive) (15) and a disease probability. The disease probability model was originally trained to distinguish eight commonest categories (7 diseases + normal lung function) detectable with full pulmonary function testing (13). For use in primary care settings, the model has been adapted to identify six categories – Asthma, COPD, interstitial lung disease (ILD), normal lung function, other obstructive disease (such as cystic fibrosis and bronchiectasis), and other unidentifiable disease – solely based on spirometry using the same training set. The rationale for reducing the number of categories to identify is based on the reduced lung function dataset available when working in a primary care environment compared to a hospital-based pulmonary function testing laboratory. The categories selected are those most commonly found in primary care, with spirometry testing indicated mainly in COPD and Asthma diagnosis.

When the quality model identifies the data quality of the spirometry session to be poor (i.e., quality of FEV1 and/or FVC is not A or B according to ATS/ERS 2019 guidelines), the following disclaimer is added to the interpretation report: "Data quality is not sufficient. Interpretation above might not represent the patient's lung function". Participants will still be asked for a diagnosis and spirometry interpretation despite the bad quality.

Reference Standard

Reference standards for diagnosis for the 50 spirometry traces were defined before commencement of the trial, by two pulmonologists. Primary and secondary care medical records were reviewed independently by two pulmonologists from the Royal Brompton & Harefield Hospitals, United Kingdom. The diagnostic reference standard was attributed to one of six categories (COPD, Asthma, ILD, OBD, Normal, Other) by each pulmonologist. In participants with multiple respiratory diagnoses (e.g. COPD and ILD), the pulmonologist was asked to choose, to the best of their abilities, the predominant category. If consensus could not be reached between the two pulmonologists, these cases and their medical records were reviewed by a third pulmonologist to adjudicate independently. All pulmonologists (SK, WM, NH) were accredited specialists in respiratory medicine with a minimum of eight years as a consultant in the National Health Service, with expertise in the diagnosis and

For peer review only - http://bmjopen.bmj.com/site/about/guidelines.xhtml

management of COPD and other chronic respiratory diseases. Pulmonologists had no access to the AI software reports, nor had any communication with the software engineer or software company. Reference standards for the technical and pattern interpretation were completed by a Respiratory Physiologist (KS) with 26 years' experience. The Respiratory Physiologist was asked to rate the technical quality of the FEV1 and FVC using the categories Acceptable (quality grade A and B ATS/ERS 2019 Guidelines) or Not Acceptable (Quality grade C D E F U)(15). For pattern interpretation of the spirometry, The Respiratory Physiologist was asked to choose from the following four categories: normal, airflow obstruction, possible restriction or non-specific pattern, possible mixed disorder. All pulmonologists and respiratory physiologist were blinded to AI software reports.

Study Outcomes

The primary endpoint for a primary care clinician participant is the performance of their preferred diagnosis, defined to be the number of correct cases identified expressed as a percentage out of the total number of spirometry records. A correct case is where the participant's preferred diagnosis matches the reference final diagnosis (described above).

The secondary endpoints, expressed per participant are listed in Table 1.

For peer review only

Table 1 Secondary endpoints

Secondary endpoints	Definition	Measured	Reference Standard
Quality assessment of the spirometry trace	A correct case is where the participant's quality grade matches the reference quality grade. Units will be percentage of total cases that are correct	Multiple choice categories: Acceptable (Grade A/B) or Not Acceptable (Grades C/D/E/F/U)	Respiratory Physiologist
Quality Assessment self-rated confidence		Visual Analogue Scale (0-10) where is 0 = not confident at all; 10= very confident)	
Pattern interpretation of the trace	A correct case is where the participants' selected pattern matches the reference pattern.<br>Units will be percentage of total cases that are correct	Multiple choice categories: Normal, Airflow obstruction, Possible restriction or non-specific pattern, Possible Mixed Disorder.	Respiratory Physiologist
Pattern interpretation self-rated confidence		Visual Analogue Scale (0-10) where is 0 = not confident at all; 10= very confident)	
Differential diagnostic performance	A correct case is where the preferred or differential diagnosis matches the reference final diagnosis. Units will be percentage of total cases that are correct	Multiple choice categories: Asthma, COPD, interstitial lung disease (ILD), normal lung function, other obstructive disease, and other unidentifiable disease	Pulmonologist
Diagnostic self-rated confidence		Visual Analogue Scale (0-10) where is 0 = not confident at all; 10= very confident)	

Sample size calculation

The sample size calculation was informed by previous feasibility data from 30 primary care practitioners assessing 5 spirometry traces. The preferred diagnosis matched the reference diagnosis in a mean (SD) of 55% (19%) of spirometry records. We have assumed the same SD of

For peer review only - http://bmjopen.bmj.com/site/about/guidelines.xhtml

19%, which is conservative because more cases will be assessed per practitioner, leading to less variable practitioner rates. As the calculation involves conservative assumptions, the power was set below 90%, to be 85%. With 132 participants (66 per group) this allows for detection of a 10% difference (a mean of 5 extra cases in 50) in the rate of cases correctly identified to match the reference standard (mean 65% versus mean 55%) using an unpaired t-test at the two-sided 5% significance level.

The recruitment target was originally estimated at 156 practitioners to allow for an estimated 15% drop out. However, a preliminary analysis of completion rate of the first 50 recruited participants demonstrated that 29 of the 50 participants scored all 50 spirometry records within the 8-week study period (58% completion). Based on this data, the study statistician advised increasing the recruitment target to 228 to account for 42% non-completion (see Amendment section).

Descriptive statistics will be performed for demographics, baseline characteristics and endpoint data: number and percentage for categorical data and mean and standard deviation or median inter-quartile range for non-normally distributed) continuous data.

Statistical Analysis

Survey responses from participants will be collated in a secured, online database. A Statistical Analysis Plan (SAP) will be developed and statistical analysis will be conducted by the study statistician. The statistician will be blinded to control/intervention group allocation. Descriptive statistics will be presented for demographic, baseline characteristics and endpoint data. Number and percentage will be presented for categorical data. Mean and standard deviation will be presented for normally distributed continuous data while median inter-quartile range will be presented for non-normally distributed continuous data.

The number of participants consented, randomised, and completing the study will be described in a study CONSORT diagram.

Data analysis will be performed on an Intention to Treat basis. Data will be analysed with the statistical software Stata version 17.0. All statistical tests will be 2-sided and significance set at $p < 0.05$.

For peer review only - http://bmjopen.bmj.com/site/about/guidelines.xhtml

Primary endpoint analysis

For each participant, diagnostic performance will be calculated as percentage of the 50 spirometry procedures where the final diagnosis was correctly identified. In each randomised group, the diagnostic performance will be summarised as the mean of the participants' diagnostic performance. A 2-sample unpaired t test will be used to assess the mean difference in correct response between the 2 randomised groups.

Randomisation will be stratified according to job role (General Practitioner: Yes or No) and Accreditation from Association for Respiratory Technology and Physiology (Performance Only, Interpretation Only, Performance and Interpretation, No accreditation), therefore an analysis of covariance will be used to compare the intervention and control groups by adjusting for the main effects of the stratified variables. To internally validate the estimate of the difference between the 2 groups nonparametric bootstrap estimation of difference will be done by taking 100 from the analysis of covariance model.

Secondary endpoint analysis

For continuous variables, the secondary endpoints will be compared between the control and intervention groups using the same approach as the primary endpoint.

Sub-group analysis

Subgroup analysis will be performed on the 20 spirometry traces where the confirmed diagnosis (reference standard) was COPD, as spirometry in primary care is most relevant to COPD diagnosis.

Missing data

Only participants who complete at least 70% of the spirometry cases will be included in the primary analysis.

Data Collection

Data will be collected via the online platform Qualtrics which is general data protection regulations (GDPR) compliant. Further information is provided at https://www.qualtrics.com/uk/platform/gdpr/.

A user license has been obtained by Royal Brompton and Harefield Hospital to use this platform for research purposes. There will be one secure user log in for one member of the study team who will be unblinded. All other members of the research team will be blinded.

For peer review only

For peer review only - http://bmjopen.bmj.com/site/about/guidelines.xhtml

Data Management

All data will be handled in accordance with the Data Protection Act (2018), NHS Caldecott Principles, The UK Policy Framework for Health and Social Care Research, and the condition of the REC approval. The online platform (Qualtrics) will be used to collect data responses from each participant reviewing the spirometry traces. Qualtrics itself will act as a database and data will also be downloaded to an excel file/csv file/database) for back up and analysis. Database access will be strictly restricted through user-specific passwords to the authorised research team members. All returned data (Microsoft Forms and Excel spreadsheets) will be held on the H drive (Harefield Hospital) by the clinical team (CI and study team) and will be password protected and only accessible by the direct study team.

No participant identifiable data, beyond basic demographics, will be transferred and all participants will have a participant identification number. Data will be downloaded typically within one week of data collection on Qualtrics by members of the research team. The research team will undertake appropriate reviews of the entered data where appropriate for the purpose of data cleaning and will request amendments as required.

At the end of the study, the CI or their assigned delegate will review all the data for each participant to verify that all the data are complete and correct. At this point, all data can be formally locked for analysis.

Patient and Public Involvement

Patient representatives are included in the trial management group and will attend the quarterly meetings across the duration of the trial. The Deputy Head of Research and Innovation for Asthma + Lung UK (the largest UK Lung Health charity) is also part of the trial management group. Our research team includes two costed co-applicants who will co-lead the PPI elements of the project.

PPI co-applicant responsibilities include: setting and refining overall PPI strategy as the project progresses, reporting on PPI activities to the research management group, evaluation, monitoring and reporting of PPI e.g. using a PPI impact log , communicating with our PPI collaborators (Asthma+ Lung UK) as well end-users (GP federations / clinical commissioning groups) on the project status, synthesising results and conclusions of PPI activities and disseminating feedback, writing up PPI

For peer review only - http://bmjopen.bmj.com/site/about/guidelines.xhtml

sections with public contributors for ethics applications, patient-facing documents and project reports

Safety Reporting

The procedure proposed for this efficacy study will not affect the usual standard of care for participants or patients. The datasets will comprise spirometry records previously collected as part of clinical spirometry pathways in primary care. As such, this retrospective analysis, which will not involve delivery of an intervention nor a change in patient's usual clinical care, is unlikely to produce direct risk for participants.

The main study risks involve patient confidentiality, information governance and avoiding bias. To address this, all data analysed by parties outside the direct clinical team will be de-identified. Spirometry records will be anonymised and provided to clinicians on an online platform (Qualtrics) compliant with GDPR.

The research team (except the platform administrator) and trial statistician will be blinded to participants' group allocation until the completion of data analysis for the primary and secondary outcomes.

Protocol amendments

Any changes to the study protocol outlined in this paper will be approved by Health Research Authority (HRA) and study Sponsor Royal Brompton and Harefield Hospitals (RBHH), Guy's and St Thomas' NHS Foundation Trust (GSTFT). Two non-substantial amendments have been submitted and approved by HRA for this study: 1. Addition of a link to the study platform in the participant information; 2. An increase in target recruitment number (detailed in Methods section).

Ethics and Dissemination

Ethical Approval

This study has been reviewed and given favourable opinion by Health Research Authority Wales (Reference: 22/HRA/5023) and registered on Clinical trials.gov (Reference: NCT05933694).

For peer review only

Dissemination

To maximise the audience for dissemination of our findings, results from the study will be disseminated by presentations at relevant scientific meetings and conferences as well as by high impact peer-reviewed publications. We will also disseminate via presentations and newsletters to participants and key stakeholders, including Asthma + Lung UK who have been collaborators on this grant. The results will be shared with local and national primary and secondary care partners and networks, including British Thoracic Society, Primary Care Respiratory Society and International Primary Care Respiratory Group. A summary report will be shared on the Royal Brompton and Harefield website research pages.

Discussion

Spirometry is a key investigation for supporting diagnosis and disease monitoring in chronic respiratory disease. There is a clear need to improve the access, quality and interpretation of spirometry in primary care and other non-respiratory community settings which have many historic challenges, further negatively impacted by the COVID pandemic (8). AI decision-support software has shown potential in improving quality assessment and interpretation of spirometry and this study will evaluate its effectiveness in interpretation of real-world spirometry results assessed by clinicians working in primary care in the UK.

This study is the first randomised controlled efficacy study with an intervention designed to improve the performance of primary care clinicians in the interpretation of spirometry using AI and an important first step for clinicians to make informed choices about its use. It will directly test the effect of an AI-powered spirometry interpretation software in primary care using real-world traces reflective of the results reviewed in practice by clinicians.

We will advertise the trial and recruit nationally to maximise participation and variation in participant sample.

Funding statement: The study is being funded by National Institute for Health Research (NIHR) through an AI Award in Health and Care (Phase 3- Application: Grant number AI_AWARD02204). The study is also supported by the NIHR Leicester Biomedical Research Centre – Respiratory theme. ST is supported by the National Institute for Health Research ARC North Thames. RR is supported by

For peer review only - http://bmjopen.bmj.com/site/about/guidelines.xhtml

the NIHR Oxford Biomedical Research Centre – Respiratory. RAE is supported by an NIHR Clinical Scientist fellowship. The views expressed in this publication are those of the authors and not necessarily those of the National Institute for Health Research or the Department of Health and Social Care.

Competing interests: WM is Honorary President of the Association for Respiratory Technology and Physiology (ARTP). MT is CEO of ArtiQ – a company that produces artificial intelligence enabled lung function support software. JM and KVO are employees of ArtiQ.

Authors' contributions: WM and MT conceived the wider research plan. All authors developed the theory and plan for this study. WB completed the statistical analysis plan. GD, EEE and WM drafted the initial manuscript. All authors reviewed, commented and approved the manuscript.

Acknowledgements: We thank our patient and public involvement members for their contribution to the overall grant application and involvement in trail management meetings. We acknowledge Asthma + Lung UK for their contribution and support with this work.

References

1. Global burden of chronic respiratory diseases and risk factors, 1990-2019: an update from the Global Burden of Disease Study 2019. EClinicalMedicine. 2023;59:101936.
2. IHME G. Global Burden of Disease Study 2019 (GBD 2019) results. Institute for Health Metrics and Evaluation Seattle, United States; 2016.
3. Jones RC, Price D, Ryan D, Sims EJ, von Ziegenweidt J, Mascarenhas L, et al. Opportunities to diagnose chronic obstructive pulmonary disease in routine care in the UK: a retrospective study of a clinical cohort. Lancet Respir Med. 2014;2(4):267-76.
4. Collard HR, Loyd JE, King TE, Jr., Lancaster LH. Current diagnosis and management of idiopathic pulmonary fibrosis: a survey of academic physicians. Respir Med. 2007;101(9):2011-6.
5. Heffler E, Crimi C, Mancuso S, Campisi R, Puggioni F, Brussino L, et al. Misdiagnosis of asthma and COPD and underuse of spirometry in primary care unselected patients. Respir Med. 2018;142:48-52.
6. NICE. Chronic obstructive pulmonary disease in over 16s: diagnosis and management 2018 [Available from: https://www.nice.org.uk/guidance/ng115.
7. NICE. Asthma: diagnosis, monitoring and chronic asthma management. 2017 [Available from: https://www.nice.org.uk/guidance/ng80.
8. Doe G, Taylor SJ, Topalovic M, Russell R, Evans RA, Maes J, et al. Spirometry services in England post-pandemic and the potential role of AI support software: a qualitative study of challenges and opportunities. British Journal of General Practice. 2023;73(737):e915-e23.
9. van de Hei SJ, Flokstra-de Blok BMJ, Baretta HJ, Doornewaard NE, van der Molen T, Patberg KW, et al. Quality of spirometry and related diagnosis in primary care with a focus on clinical use. NPJ Prim Care Respir Med. 2020;30(1):22.
10. Hegewald MJ, Gallo HM, Wilson EL. Accuracy and Quality of Spirometry in Primary Care Offices. Ann Am Thorac Soc. 2016;13(12):2119-24.

For peer review only - http://bmjopen.bmj.com/site/about/guidelines.xhtml

11. Societies FoIR. The global impact of respiratory disease. Third Edition. European Respiratory Society. 2021 2021.
12. Das N, Verstraete K, Stanojevic S, Topalovic M, Aerts J-M, Janssens W. Deep-learning algorithm helps to standardise ATS/ERS spirometric acceptability and usability criteria. European Respiratory Journal. 2020;56(6):2000603.
13. Topalovic M, Das N, Burgel PR, Daenen M, Derom E, Haenebalcke C, et al. Artificial intelligence outperforms pulmonologists in the interpretation of pulmonary function tests. Eur Respir J. 2019;53(4).
14. Desbordes P, Topole E, Biondaro S, Montagna I, Corre S, Topalovic M. AI Over-reading Based on ATS/ERS 2019 Criteria Is a Reliable Option for Instant Spirometry Quality Control in Clinical Trials. B70 INHALE THE FUTURE: NEW FINDINGS IN PULMONARY FUNCTION. p. A4064-A.
15. Graham BL, Steenbruggen I, Miller MR, Barjaktarevic IZ, Cooper BG, Hall GL, et al. Standardization of Spirometry 2019 Update. An Official American Thoracic Society and European Respiratory Society Technical Statement. American Journal of Respiratory and Critical Care Medicine. 2019;200(8):e70-e88.

For peer review only

Supplement_Comparing performance of primary care clinicians in the interpretation of SPIROmetry with or without Artificial Intelligence Decision support software (SPIRO-AID): a protocol for a randomised controlled trial.

Supplement

Clinician letter (participant information)

Royal Brompton and Harefield

NHS Guy's and St Thomas' NHS Foundation Trust

IRAS ID: 323361

Invitation to participate in research study: A randomized controlled trial comparing diagnostic performance of primary care clinicians in the interpretation of **SPIRO**metry with or without **A**rtificial **I**ntelligence **D**ecision support software (SPIRO-AID)

Dear Clinician,

We would like to invite you to take part in our research study. This study aims to evaluate whether an artificial intelligence decision support software (ArtiQ. Spiro) improves the diagnostic accuracy of spirometry interpreted by primary care clinicians, as measured by Clinician Diagnostic Accuracy (*vs* Reference Standard). You are being invited to take part because you are a clinician working in primary care who refers patients for spirometry and/or receives spirometry results for review.

The study is being conducted by Harefield Hospital, part of Guy's and St Thomas's NHS Foundation Trust (GSTFT), in partnership with ArtiQ, a company specialising in respiratory medicine and Artificial Intelligence (AI). The Chief Investigator (CI) is Prof William Man, Professor and Honorary Consultant Respiratory Physician. The study is being funded by National Institute for Health Research (NIHR) through an AI Award in Health and Care (Phase 3- Application: Grant number AI_AWARD02204). The lead organisation for the wider research project is Guy's and St Thomas's NHS Foundation Trust. The study is also supported by the NIHR Leicester Biomedical Research Centre - Respiratory.

Please take time to read the following information carefully. Please discuss it with others, including your colleagues if you wish. Please ask us if there is anything that is not clear.

Purpose of the Study: The NHS Long Term Plan (LTP) has identified quality assured spirometry as a clinical priority through investment in training more community staff to perform and interpret spirometry more accurately. However, this process takes time and disagreement over spirometry interpretation can still occur between trained staff. The purpose of this study is to evaluate whether an AI decision support software improves the diagnostic accuracy and quality assessment of spirometry by primary care clinicians.

Taking part: If you are happy to take part in this research please click here where you will be provided with a link to an on-line platform (Qualtrics) to assess your eligibility in the study and to complete a Consent form. You will then be asked about your job role and any previous experience with spirometry, which will help the study team to randomise participants into each arm of the study. Some participants will be randomised to receive

SPIRO-AID Clinician Information Letter
Version [illegible]
Page 1 of 3

Supplement_Comparing performance of primary care clinicians in the interpretation of SPIROmetry with or without Artificial Intelligence Decision support software (SPIRO-AID): a protocol for a randomised controlled trial.

50 spirometry traces for review in the usual format that they would be received. Some participants will be randomised to receive 50 spirometry traces plus an AI report (ArtiQ.Spiro). The 50 spirometry traces will be from a de-identified retrospective dataset. We anticipate that reviewing the traces will take approximately one hour of your time.

Expenses and payments: Participants will be offered reimbursement (£150) for their time. Reimbursement details are provided via the Qualtrics study platform after you have completed review of the 50 traces.

Risks and benefits of taking part: The procedure proposed for this study does not affect the usual standard of care for participants. The datasets will comprise spirometry data previously collected as part of clinical spirometry pathways in primary care. As such, this retrospective data analysis, which does not involve delivery of an intervention nor a change in patient's usual clinical care, is unlikely to produce direct risk for participants. This study will help us to understand if the addition of AI improves diagnostic accuracy and quality assessment of spirometry, informing design of spirometry pathways for the future.

Study period: The study period will be six months. When this period is over, the results will be analysed and written up for dissemination. The results will be presented at scientific meetings and will be published in medical journals. A dissemination event may be arranged and we will invite participants to attend.

Confidentiality: All information which is collected about you during the course of the research will be kept strictly confidential. Royal Brompton and Harefield Hospitals, part of Guy's and St Thomas NHS Foundation Trust is the sponsor for this study. The sponsor will act as the data controller. This means that it is responsible for looking after your information and using it properly. A web based electronic platform (Qualtrics) will be used to collect data responses from each participant reviewing the spirometry traces.

How will we use information about you?

We will need to use information from you for this research project. This information will include your name and contact details. People will use this information to do the research or to check your records to make sure that the research is being done properly.

People who do not need to know who you are will not be able to see your name or contact details. Your data will have a code number instead. We will keep all information about you safe and secure. Database access will be strictly restricted through user-specific passwords to the authorised research team members. At the end of the study, the CI or their assigned delegate will review all the data for each participant to verify that all the data are complete and correct. At this point, all data can be formally locked for analysis.

Supplement_Comparing performance of primary care clinicians in the interpretation of SPIROmetry with or without Artificial Intelligence Decision support software (SPIRO-AID): a protocol for a randomised controlled trial.

Once we have finished the study, we will keep some of the data so we can check the results. We will write our reports in a way that no-one can work out that you took part in the study.

What are your choices about how your information is used?
You can stop being part of the study at any time, without giving a reason, but we will keep information about you that we already have.
We need to manage your records in specific ways for the research to be reliable. This means that we won't be able to let you see or change the data we hold about you.

Where can you find out more about how your information is used?
You can find out more about how we use your information

- at www.hra.nhs.uk/information-about-patients/
- by asking one of the research team
- by sending an email to ig@rbht.nhs.uk, or
- by ringing us on 0207 352 8121 ext. 2610.

Additional information on the use of patient data in research in line with the General Data Protection Regulation (GDPR) is also provided here.

If you have a concern about any aspect of this study you should ask to speak with the researchers who will do their best to answer your questions [Insert local contact number]. If you remain unhappy and wish to complain formally, you can do this. Details can be obtained from the Royal Brompton and Harefield Hospitals (RBHH) Research Office *via* Email: (Insert)

Further Information and Contact Details:
If you require any further information, please do not hesitate to contact us on the details below.

Study email: Insert

Royal Brompton and Harefield Hospital Research Team
Contact [Insert TBC]

Gillian Doe (Research Programme Manager)
Respiratory BRC, Leicester **Tel:** 0116 258 3370 **Email:** ged6@leicester.ac.uk

Yours faithfully,

[Insert signature]
Professor Will Man (CI)

Supplement_Comparing performance of primary care clinicians in the interpretation of SPIROmetry with or without Artificial Intelligence Decision support software (SPIRO-AID): a protocol for a randomised controlled trial.

Consent form on Study platform

Q15

Please initial each box if you are in agreement

	Initials
I confirm that I have read and understand the information in the Clinician invitation letter dated 07/03/2023 (Version 1.2) for the above study and have had the opportunity to consider the information, ask questions and have these answered satisfactorily.	
I understand that my participation is voluntary and that I am free to withdraw at any time, without giving any reason, without medical care or legal rights being affected.	
I understand that data collected during the study may be looked at by responsible individuals from the study Sponsor or from regulatory authorities where it is relevant to my taking part in research. I give permission to these individuals to have access this data.	
I agree with the publication of the results of this study in a medical journal (all data will be published anonymously).	
I agree to take part in the above study.	

Q16

Name of Participant

Q17

Date

${date://CurrentDate/FL}

Q21

Type your full name below in lieu of a signature

For peer review only - http://bmjopen.bmj.com/site/about/guidelines.xhtml

Supplement_Comparing performance of primary care clinicians in the interpretation of SPIROmetry with or without Artificial Intelligence Decision support software (SPIRO-AID): a protocol for a randomised controlled trial.

Example AI-decision support software report

ARTIQ

Session ID: 120519_1987
Analysed on: 2023-03-23 14:50:25 UTC±00:00

Session Evaluation

FEV1 (Grade A) Acceptable
FVC (Grade B) Acceptable

Best Trial Results
Best Trial: 1
FEV1: 0.7 L
FVC: 2.19 L

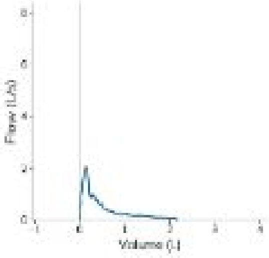

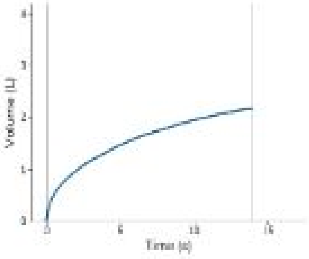

Trial Overview

	FEV1		FVC	
	Acceptable	Usable	Acceptable	Usable
Trial 1	Yes	-	Yes	-
Trial 2	Yes	-	Yes	-
Trial 3	Yes	-	No	Yes

Trial Evaluation Details

TRIAL 1
FEV1 Acceptable
FVC Acceptable

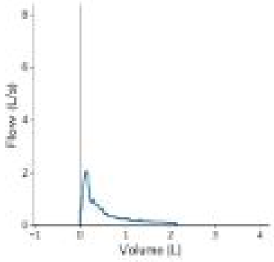

No artefact on MEFVC

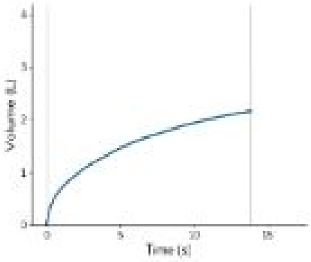

BEV Criteria: True
EOFE Criteria: True
Plateau Criteria: False
(39.35 mL change in last second)
tFE > 15s: False
FVC Repeatable: True
(30.34 mL lower than max acceptable FVC)
FIVC-FVC Criteria: None

Warnings
No second inspiration

For peer review only - http://bmjopen.bmj.com/site/about/guidelines.xhtml

Supplement_Comparing performance of primary care clinicians in the interpretation of SPIROmetry with or without Artificial Intelligence Decision support software (SPIRO-AID): a protocol for a randomised controlled trial.

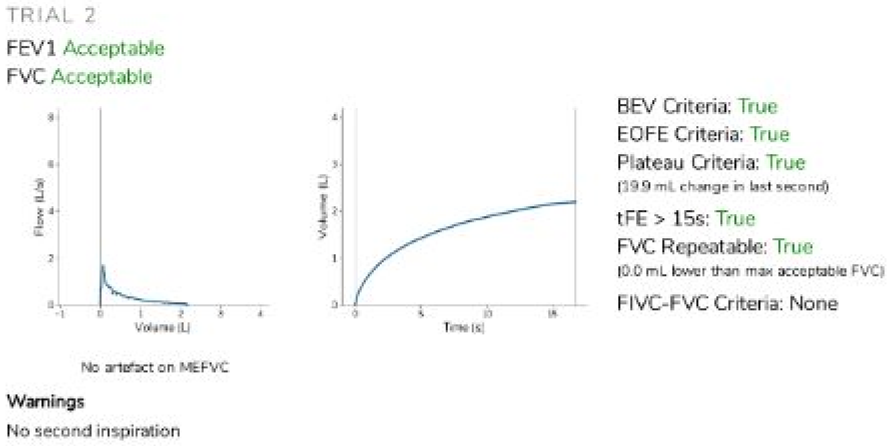

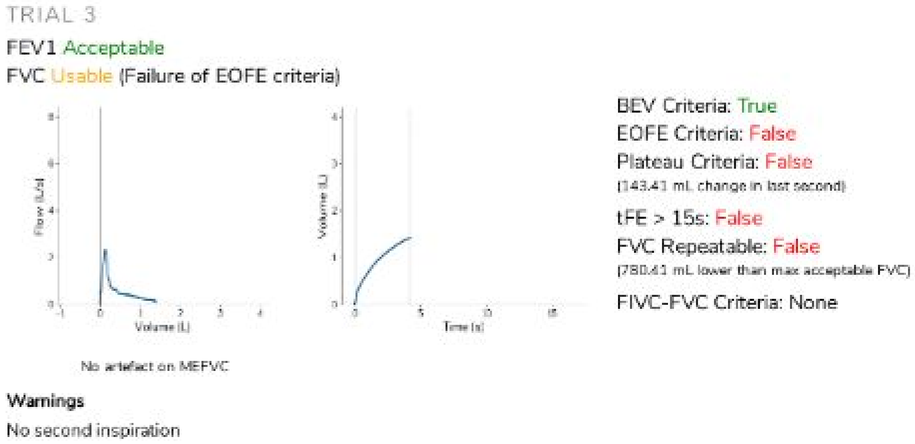

*Note no second inspiration present on these curves as this is a retrospective dataset where second inspiration was not captured

For peer review only - http://bmjopen.bmj.com/site/about/guidelines.xhtml

Supplement_Comparing performance of primary care clinicians in the interpretation of SPIROmetry with or without Artificial Intelligence Decision support software (SPIRO-AID): a protocol for a randomised controlled trial.

ARTIQ

Analyzed: 2023-04-18 23:33 UTC±00:00

Report ID: _120519_1987

Age: 53 **Gender:** Female **Current Smoker:** Yes **Pack-Years:** N/A

Interpretation of lung function tests

Spirometry demonstrates an obstructive pattern, but a restrictive component is not excluded.

Disease probability

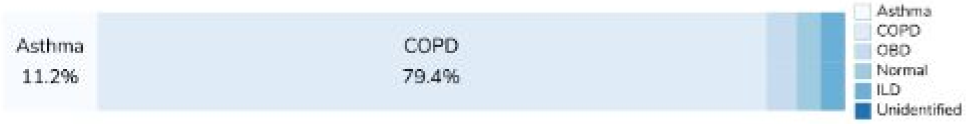

Conclusions and suggestions

Highest disease probability based on lung function: **COPD**

Consider referring to specialist and/or further testing as appropriate:
Careful auscultation on all lung areas for presence of rhonchi. Repeat spirometry with bronchodilator test. Perform screening laboratory (including eosinophilia). Perform X-ray of the thorax. Consider bronchodilator therapy.

Warnings

Probability of disease presence may not be accurate due to missing correct information of pack-years.

Legend

COPD	Chronic Obstructive Pulmonary Disease
OBD	Other Obstructive Diseases (including: cystic fibrosis, bronchiectasis, bronchiolitis)
Normal	Normal lung function
ILD	Interstitial lung disease (including: idiopathic pulmonary fibrosis, nonspecific interstitial pneumonitis, sarcoidosis)
Unidentified	Unidentified (including: neuromuscular disease, pulmonary vascular disease, thoracic deformity, pleural disease)

This report is approved for clinical use in the EU.
Automatically generated by ArtiQ.PFT VERSION NOT SET • Manufactured by ArtiQ NV • Leuven, Belgium

info@ArtiQ.eu
www.ArtiQ.eu

SPIRIT
STANDARD PROTOCOL ITEMS: RECOMMENDATIONS FOR INTERVENTIONAL TRIALS

SPIRIT 2013 Checklist: Recommended items to address in a clinical trial protocol and related documents*

Section/item	ItemNo	Description	Page no.
**Administrative information**			
Title	1	Descriptive title identifying the study design, population, interventions, and, if applicable, trial acronym	1
Trial registration	2a	Trial identifier and registry name. If not yet registered, name of intended registry	2<br>Clinical trials.gov (Reference: NCT05933694).
	2b	All items from the World Health Organization Trial Registration Data Set	
Protocol version	3	Date and version identifier	Version 1 for Publication
Funding	4	Sources and types of financial, material, and other support	18<br>Funded by National Institute for Health Research (NIHR) through an AI Award in Health and Care (Phase 3-Application: Grant number AI_AWARD02204
Roles and responsibilities	5a	Names, affiliations, and roles of protocol contributors	1
	5b	Name and contact information for the trial sponsor	4<br>Royal Brompton and Harefield Hospitals (RBHH), Guy's and St Thomas' NHS Foundation Trust (GSTFT)

For peer review only

	5c	Role of study sponsor and funders, if any, in study design; collection, management, analysis, and interpretation of data; writing of the report; and the decision to submit the report for publication, including whether they will have ultimate authority over any of these activities	18
	5d	Composition, roles, and responsibilities of the coordinating centre, steering committee, endpoint adjudication committee, data management team, and other individuals or groups overseeing the trial, if applicable (see Item 21a for data monitoring committee)	18
**Introduction**			
Background and rationale	6a	Description of research question and justification for undertaking the trial, including summary of relevant studies (published and unpublished) examining benefits and harms for each intervention	3 and 4
	6b	Explanation for choice of comparators	
Objectives	7	Specific objectives or hypotheses	4
Trial design	8	Description of trial design including type of trial (eg, parallel group, crossover, factorial, single group), allocation ratio, and framework (eg, superiority, equivalence, noninferiority, exploratory)	4
**Methods: Participants, interventions, and outcomes**			

For peer review only

Study setting	9	Description of study settings (eg, community clinic, academic hospital) and list of countries where data will be collected. Reference to where list of study sites can be obtained	6
Eligibility criteria	10	Inclusion and exclusion criteria for participants. If applicable, eligibility criteria for study centres and individuals who will perform the interventions (eg, surgeons, psychotherapists)	5
Interventions	11a	Interventions for each group with sufficient detail to allow replication, including how and when they will be administered	10 and 11
	11b	Criteria for discontinuing or modifying allocated interventions for a given trial participant (eg, drug dose change in response to harms, participant request, or improving/worsening disease)	
	11c	Strategies to improve adherence to intervention protocols, and any procedures for monitoring adherence (eg, drug tablet return, laboratory tests)	
	11d	Relevant concomitant care and interventions that are permitted or prohibited during the trial	n/a
Outcomes	12	Primary, secondary, and other outcomes, including the specific measurement variable (eg, systolic blood pressure), analysis metric (eg, change from baseline, final value, time to event), method of aggregation (eg, median, proportion), and time point for each outcome. Explanation of the clinical relevance of chosen efficacy and harm outcomes is strongly recommended	12,13

For peer review only - http://bmjopen.bmj.com/site/about/guidelines.xhtml

Participant timeline	13	Time schedule of enrolment, interventions (including any run-ins and washouts), assessments, and visits for participants. A schematic diagram is highly recommended (see Figure)	10<br>Figure 2 – study schedule
Sample size	14	Estimated number of participants needed to achieve study objectives and how it was determined, including clinical and statistical assumptions supporting any sample size calculations	13 and 14
Recruitment	15	Strategies for achieving adequate participant enrolment to reach target sample size	14
**Methods: Assignment of interventions (for controlled trials)**			
Allocation:			
Sequence generation	16a	Method of generating the allocation sequence (eg, computer-generated random numbers), and list of any factors for stratification. To reduce predictability of a random sequence, details of any planned restriction (eg, blocking) should be provided in a separate document that is unavailable to those who enrol participants or assign interventions	5
Allocation concealment mechanism	16b	Mechanism of implementing the allocation sequence (eg, central telephone; sequentially numbered, opaque, sealed envelopes), describing any steps to conceal the sequence until interventions are assigned	
Implementation	16c	Who will generate the allocation sequence, who will enrol participants, and who will assign participants to interventions	5 and 6

Blinding (masking)	17a	Who will be blinded after assignment to interventions (eg, trial participants, care providers, outcome assessors, data analysts), and how	6, 12, 14 and 15
	17b	If blinded, circumstances under which unblinding is permissible, and procedure for revealing a participant's allocated intervention during the trial	
**Methods: Data collection, management, and analysis**			
Data collection methods	18a	Plans for assessment and collection of outcome, baseline, and other trial data, including any related processes to promote data quality (eg, duplicate measurements, training of assessors) and a description of study instruments (eg, questionnaires, laboratory tests) along with their reliability and validity, if known. Reference to where data collection forms can be found, if not in the protocol	15 and 16
	18b	Plans to promote participant retention and complete follow-up, including list of any outcome data to be collected for participants who discontinue or deviate from intervention protocols	9 and 10<br>Figure 2
Data management	19	Plans for data entry, coding, security, and storage, including any related processes to promote data quality (eg, double data entry; range checks for data values). Reference to where details of data management procedures can be found, if not in the protocol	15 and 16

Statistical methods	20a	Statistical methods for analysing primary and secondary outcomes. Reference to where other details of the statistical analysis plan can be found, if not in the protocol	13 and 14
	20b	Methods for any additional analyses (eg, subgroup and adjusted analyses)	15
	20c	Definition of analysis population relating to protocol non-adherence (eg, as randomised analysis), and any statistical methods to handle missing data (eg, multiple imputation)	15
**Methods: Monitoring**			
Data monitoring	21a	Composition of data monitoring committee (DMC); summary of its role and reporting structure; statement of whether it is independent from the sponsor and competing interests; and reference to where further details about its charter can be found, if not in the protocol. Alternatively, an explanation of why a DMC is not needed	15 and 16 – data management.<br>No DMC<br>Data will be monitored, checked and cleaned by the study team.
	21b	Description of any interim analyses and stopping guidelines, including who will have access to these interim results and make the final decision to terminate the trial	15
Harms	22	Plans for collecting, assessing, reporting, and managing solicited and spontaneously reported adverse events and other unintended effects of trial interventions or trial conduct	16 and 17

Auditing	23	Frequency and procedures for auditing trial conduct, if any, and whether the process will be independent from investigators and the sponsor	The Sponsor operates a risk based monitoring and audit programme, to which this study will be subject.
**Ethics and dissemination**			
Research ethics approval	24	Plans for seeking research ethics committee/institutional review board (REC/IRB) approval	2<br>This study has been reviewed and given favourable opinion by Health Research Authority Wales (Reference: 22/HRA/5023).
Protocol amendments	25	Plans for communicating important protocol modifications (eg, changes to eligibility criteria, outcomes, analyses) to relevant parties (eg, investigators, REC/IRBs, trial participants, trial registries, journals, regulators)	17
Consent or assent	26a	Who will obtain informed consent or assent from potential trial participants or authorised surrogates, and how (see Item 32)	5 and 6
	26b	Additional consent provisions for collection and use of participant data and biological specimens in ancillary studies, if applicable	n/a
Confidentiality	27	How personal information about potential and enrolled participants will be collected, shared, and maintained in order to protect confidentiality before, during, and after the trial	Supplement<br>- Clinician letter (participant information sheet)
Declaration of interests	28	Financial and other competing interests for principal investigators for the overall trial and each study site	18

For peer review only

Access to data	29	Statement of who will have access to the final trial dataset, and disclosure of contractual agreements that limit such access for investigators	
Ancillary and post-trial care	30	Provisions, if any, for ancillary and post-trial care, and for compensation to those who suffer harm from trial participation	Participation is considered to be very low risk and does not involve patients.
Dissemination policy	31a	Plans for investigators and sponsor to communicate trial results to participants, healthcare professionals, the public, and other relevant groups (eg, via publication, reporting in results databases, or other data sharing arrangements), including any publication restrictions	17
	31b	Authorship eligibility guidelines and any intended use of professional writers	
	31c	Plans, if any, for granting public access to the full protocol, participant-level dataset, and statistical code	
**Appendices**			
Informed consent materials	32	Model consent form and other related documentation given to participants and authorised surrogates	Supplement
Biological specimens	33	Plans for collection, laboratory evaluation, and storage of biological specimens for genetic or molecular analysis in the current trial and for future use in ancillary studies, if applicable	n/a

*It is strongly recommended that this checklist be read in conjunction with the SPIRIT 2013 Explanation & Elaboration for important clarification on the items. Amendments to the protocol should be tracked and dated. The SPIRIT checklist is copyrighted by the SPIRIT Group under the Creative Commons "Attribution-NonCommercial-NoDerivs 3.0 Unported" license.

For peer review only - http://bmjopen.bmj.com/site/about/guidelines.xhtml

BMJ Open

Comparing performance of primary care clinicians in the interpretation of SPIROmetry with or without Artificial Intelligence Decision support software (SPIRO-AID): a protocol for a randomised controlled trial.

Journal:	*BMJ Open*
Manuscript ID	bmjopen-2024-086736.R1
Article Type:	Protocol
Date Submitted by the Author:	16-May-2024

Complete List of Authors:	Doe, Gillian; University of Leicester, NIHR Biomedical Research Centre<br>El-Emir, E; Guy's and St Thomas' NHS Foundation Trust<br>Edwards, G; Guy's and St Thomas' NHS Foundation Trust<br>Topalovic, Marko; ARTIQ<br>Evans , Rachael; University of Leicester, NIHR Biomedical Research Centre<br>Russell, Richard; King's College London, Kings Centre for Lung Health<br>Sylvester, Karl; Cambridge University Hospitals NHS Foundation Trust, Cambridge Respiratory Physiology<br>Orshoven, K; ARTIQ<br>Sunjaya, A; The George Institute for Global Health; Guy's and St Thomas' Hospitals NHS Trust<br>Scott, D; University of Southampton, Southampton Health Technology Assessments Centre<br>Prevost, A Toby; King's College London, Nightingale-Saunders Clinical Trials and Epidemiology Unit<br>Harvey, J; Guy's and St Thomas' Hospitals NHS Trust<br>Taylor, Stephanie; Queen Mary University of London, Wolfson Institute of Population Health<br>Hopkinson, Nicholas; Imperial College London, National Heart and Lung Insititute<br>Kon, S; Hillingdon Hospitals NHS Foundation Trust, Department of Respiratory Medicine; Guy's and St Thomas' NHS Foundation Trust, Department of Respiratory Medicine<br>Jarrold, I; Asthma and Lung UK<br>Spain, N; PPI partner<br>Banya, W; Guy's and St Thomas' Hospitals NHS Trust<br>Man, William; Guy's and St Thomas' Hospitals NHS Trust; King's College London, King's Centre for Lung Health

**Primary Subject Heading**:	Respiratory medicine
Secondary Subject Heading:	Diagnostics, General practice / Family practice
Keywords:	RESPIRATORY MEDICINE (see Thoracic Medicine), Primary Care < Primary Health Care, Lung Diseases

Title: Comparing performance of primary care clinicians in the interpretation of SPIROmetry with or without Artificial Intelligence Decision support software (SPIRO-AID): a protocol for a randomised controlled trial.

Authors:

Doe G[1], El-Emir E[2], Edwards G[2], Topalovic M[3], Evans RA[1], Russell R[4], [3], Sylvester KP[5], Van Orshoven K[3], Sunjaya AP[2,6], Scott DA[7], Prevost AT[8], Harvey J[2], Taylor SJC[9], Hopkinson NS[10], Kon SS[2,11], Jarrold I[12], Spain N[13], Banya W[2,14] and Man WD-C[2, 4, 10]

Institutions:

1. NIHR Biomedical Research Centre (Respiratory Theme), Department of Respiratory Science, University of Leicester, Leicester, UK
2. Harefield Respiratory Research Group, Royal Brompton & Harefield Hospitals, Guy's and St Thomas' NHS Foundation Trust, UK
3. ARTIQ, Leuven, Belgium
4. King's Centre for Lung Health, Faculty of Life Sciences and Medicine, King's College, London.
5. Royal Papworth Hospital NHS Foundation Trust, UK
6. The George Institute for Global Health, UNSW Sydney, Australia and The George Institute for Global Health, Imperial College London, UK
7. Southampton Health Technology Assessments Centre, University of Southampton, Southampton, UK; Diabetes Research Centre, University of Leicester, Leicester, UK
8. Nightingale-Saunders Clinical Trials and Epidemiology Unit, Cicely Saunders Institute of Palliative Care, Policy and Rehabilitation, Kings College London, UK
9. Wolfson Institute of Population Health, Queen Mary University of London, London, UK
10. National Heart & Lung Institute, Imperial College London, UK
11. Department of Respiratory Medicine, The Hillingdon Hospitals NHS Foundation Trust, UK
12. Asthma + Lung UK
13. Patient and public involvement representative, UK
14. Medical Statistics, Research & Development, Royal Brompton & Harefield Hospitals, Guy's and St Thomas' NHS Foundation Trust, UK

Author for correspondence: Gillian Doe, Leicester NIHR Biomedical Research Centre – Respiratory, Glenfield Hospital, Leicester LE3 9QP

Email: ged6@leicester.ac.uk ORCID ID: 0000-0003-4782-581

For peer review only - http://bmjopen.bmj.com/site/about/guidelines.xhtml

Abstract

Introduction: Spirometry is a point-of-care lung function test that helps support the diagnosis and monitoring of chronic lung disease. The quality and interpretation accuracy of spirometry is variable in primary care. This study aims to evaluate whether artificial intelligence (AI) decision support software improves the performance of primary care clinicians in the interpretation of spirometry, against reference standard (expert interpretation)

Methods and Analysis: A parallel, two-group, statistician-blinded, randomised controlled trial of primary care clinicians in the United Kingdom, who refer for, or interpret, spirometry. People with specialist training in respiratory medicine to consultant level were excluded. A minimum target of 228 primary care clinician participants will be randomised with a 1:1 allocation to assess fifty de-identified, real-world patient spirometry sessions through an on-line platform either with (Intervention group) or without (Control group) AI decision support software report. Outcomes will cover primary care clinicians' spirometry interpretation performance including measures of technical quality assessment, spirometry pattern recognition and diagnostic prediction, compared with reference standard. Clinicians' self-rated confidence in spirometry interpretation will also be evaluated. The primary outcome is the proportion of the fifty spirometry sessions where the participant's preferred diagnosis matches the reference diagnosis. Unpaired t-tests and Analysis of Covariance will be used to estimate the difference in primary outcome between intervention and control groups.

Ethics and Dissemination: This study has been reviewed and given favourable opinion by Health Research Authority Wales (Reference: 22/HRA/5023) and registered on Clinical trials.gov (Reference: NCT05933694).

Results will be submitted for publication in peer-reviewed journals, presented at relevant national and international conferences, disseminated through social media, patient and public routes and directly shared with stakeholders.

Trial registration: NCT05933694

For peer review only

For peer review only - http://bmjopen.bmj.com/site/about/guidelines.xhtml

Strengths and Limitations:

- Real-world spirometry traces completed in primary care, irrespective of technical quality, will be used.
- To replicate real-world primary care in the United Kingdom, study participants will not be limited to general practitioners, but will include other members of multidisciplinary team that are expected to perform or/and interpret spirometry in primary care.
- The trial will be advertised and recruited nationally to maximise participation and variation in participant sample.
- The trial statistician will be blinded to group allocation.
- Trial outcomes will include all relevant aspects of spirometry interpretation, including assessment of technical quality, pattern recognition and diagnosis prediction.

Introduction

Chronic respiratory disease has significant negative impact on quality of life and is the third leading cause of death globally, and one of the highest contributors to economic burden for healthcare systems (1). Chronic Obstructive Pulmonary Disease (COPD) alone accounted for 3.3 million deaths worldwide in 2019, increasing 14% over the previous ten years (2). Accurate and timely diagnosis of respiratory disease is key to improving access to treatment and patient outcomes, and long delays to diagnosis for individuals with chronic lung diseases are well documented (3-5).

Spirometry is a simple, point-of-care lung function procedure recommended to support the diagnosis and monitoring of COPD and asthma, the commonest long-term respiratory conditions (6, 7). In many western and low and middle income (LMIC) countries, spirometry will be performed and interpreted in non- specialist respiratory settings. However, there is inequity in spirometry provision in primary care and community settings; historical challenges with quality, skills and competence in interpretation, and funding, have been compounded by the COVID-19 pandemic (8). Spirometry

For peer review only - http://bmjopen.bmj.com/site/about/guidelines.xhtml

performed in primary care and community settings has been shown to not meet international criteria in over 85% of cases (9). A low level of confidence in assessing spirometry quality is also common amongst general practitioners (10). Improving prevention and early diagnosis has been identified as a priority in the Global Impact of Respiratory Disease report (11). In practice, this requires improved equity, access and quality of spirometry provision.

Recent work has shown that Artificial Intelligence (AI)–powered decision support software can provide automated technical quality assessment and interpretation of full lung function tests (12, 13). This software can outperform pulmonologists in diagnostic prediction from lung function tests (13). Similar AI software that interprets spirometry (usually the only lung function test accessible in many healthcare settings) may be particularly helpful in primary care to support clinicians who have generalist roles and less experience in respiratory diagnosis. Qualitative work highlighted that validation and evaluation of the effectiveness of AI software is key in engaging clinicians and commissioners to have confidence in implementation in clinical practice.

The primary objective of this study is to compare the performance of primary care clinicians in the interpretation of 50 real-world spirometry records with or without AI spirometry decision support software reports.

Methods and Analysis

Trial Design and Registration

This is a parallel, two group, randomised controlled, statistician-blinded efficacy trial to evaluate whether AI spirometry decision support software can improve the performance of primary care clinicians in their interpretation of real-world spirometry. Each clinician will be provided with the same clinical dataset of 50 de-identified, real-world patient spirometry records through an online platform (Qualtrics XM), and asked to answer questions about preferred diagnosis (i.e. diagnosis they think is most likely) and differential diagnosis (i.e. second most likely diagnosis) based on spirometry and limited clinical data, the technical quality of spirometry and the spirometry pattern. Questions will also explore clinicians' confidence in their assessments.

For peer review only - http://bmjopen.bmj.com/site/about/guidelines.xhtml

Participants will be allocated at random to receive either spirometry records alone or spirometry records with the addition of the AI software report. The clinical spirometry records will be de-identified (name, date of birth, address, postcode, occupation, GP, medications data removed), by a member of the clinical care team.

Royal Brompton and Harefield Hospitals (RBHH), Guy's and St Thomas' NHS Foundation Trust (GSTFT) will act as study sponsor and the trial has been registered with clinicaltrials.gov (NCT05933694). This study has been reviewed and given favourable opinion by Health Research Authority Wales (Reference: 22/HRA/5023).

Study Population

Potential participants will be clinicians working in primary care involved in spirometry referral and interpretation.

Eligibility Criteria

Eligibility criteria will be: 1) A clinician working in primary care (defined as >50% of job plan in primary care) in the United Kingdom who refers for or performs spirometry (typically GP, practice nurse); 2) Able to access an online study platform in order to review spirometry sessions; 3) Able to provide informed consent via the study platform.

Exclusion criterion will be: 1) Clinicians who have completed specialist training in respiratory medicine and are recognised by the General Medical Council with the right to practise as a consultant in respiratory medicine in the National Health Service.

Eligibility criteria will be used as screening questions on the on-line study platform prior to participants being able to consent to participate.

Participants will be randomised 1:1 to either control (report 50 spirometry records alone) or intervention (report same 50 spirometry records with access to an AI spirometry interpretation report for each spirometry result). Randomisation will be done by the study on-line platform at the point of consent to the study by minimising according to job role (General Practitioner: Yes or No) and spirometry accreditation from the Association for Respiratory Technology and Physiology (Performance Only, Interpretation Only, Performance and Interpretation, No accreditation) and then randomising by a random number generation algorithm performed by the on-line platform.

For peer review only - http://bmjopen.bmj.com/site/about/guidelines.xhtml

Recruitment and Consent

Recruitment to the study will be advertised through national and local primary care networks, professional networks, the institution website and social media will be used. By advertising and recruiting nationally, the recruitment strategy aims to engage with clinicians from diverse backgrounds in terms of the patient population they serve, age, years of clinical experience, job role and ethnicity. We will offer financial reimbursement for all potential participants participating and completing the study, which may attract some who might not be interested in AI or respiratory diagnostics.

Clinicians will be sent a letter outlining the research study with the option for them to express interest in participating. The letter serves as the participant information sheet (see supplementary information) and will be sent via email. Participants who express interest in taking part will be sent a link to the on-line study platform where they will be able to complete the eligibility screening questions and, if applicable, consent form online. The study will also be advertised on the Royal Brompton and Harefield Hospitals Research website with a link available to the on-line study platform. Participants will be able to withdraw without giving any reason, and the number of participants registering but not completing the study will be recorded. Randomisation will be kept concealed until the point of the allocation of the participant to a study arm.

Setting

The study will be conducted via an online platform (Qualtrics XM).

Procedure

Study participants (participating clinicians) will independently assess the same 50 spirometry records, in the same order, through a bespoke designed online platform developed using Qualtrics XM (Seattle, USA). Participants will be blinded to the reference standards. Please see Figure 1 for example. The patient spirometry records were randomly selected from a database comprising 1122 patients undergoing spirometry in primary care and community-based respiratory clinics in Hillingdon borough between 2015-2019. An online randomizer will be used to select the 50 spirometry sessions used in the trial (https://www.randomizer.org/), according to “disease category” so that of the 50 spirometry traces selected, 40% will be from patients with COPD, 20% will have

For peer review only - http://bmjopen.bmj.com/site/about/guidelines.xhtml

Normal spirometry, and 10% for each of the four other disease categories (Asthma, ILD, Other obstructive, Other disease/unidentifiable category), to represent a likely distribution of diagnoses in a primary care setting.

For each spirometry record, the primary care clinician participant will answer the following questions on the Qualtrics platform:

- What is the Technical Quality of the spirometry? (a drop-down box containing the options: Acceptable (Quality grade A or B), Not acceptable). Technical quality will be defined according to the ATS/ERS 2019 Spirometry Technical Statement, provided to participants via a link embedded in the on-line platform
    - One response for Forced expiratory volume in one second (FEV_1) and one question for Forced Vital Capacity (FVC)
- How confident are you in your technical quality assessment using a visual analogue scale (0: Not confident at all to 10: Very Confident)?
- Provide a pattern interpretation based on spirometry report received. Options: Normal, Airflow Obstruction, Possible restriction or non-specific pattern, Possible mixed disorder
- How confident are you in your pattern interpretation using a visual analogue scale (0: Not confident at all to 10: Very Confident)?
- Provide a preferred diagnosis based on spirometry report received and information. (a drop-down box containing the options: Asthma, COPD, interstitial lung disease (ILD), normal lung function, other obstructive disease, and other unidentifiable disease): **Preferred Diagnosis**
- Provide a differential diagnosis based on spirometry report received and information. (a drop-down box containing the options: Asthma, COPD, interstitial lung disease (ILD), normal lung function, other obstructive disease, and other unidentifiable disease)**: Differential Diag**nosis. The previously chosen 'preferred diagnosis' is automatically removed from the list of available options.
- How confident are you in your diagnosis using a visual analogue scale (0: Not confident at all to 10: Very Confident)

For peer review only - http://bmjopen.bmj.com/site/about/guidelines.xhtml

Participants will have eight weeks to review the 50 spirometry traces; reminders will be sent at weeks three, six and seven to those who have not completed their assessments. (Figure 2).

Intervention – AI spirometry software

The AI software was developed by ArtiQ (Leuven, Belgium), and provides AI-supported quality assessment and interpretation guidance of spirometry sessions (Supplement). The quality assessment component leverages deep learning methods to perform the subjective assessment of spirometry quality assessment (i.e., related to curve shape), as well as implementing the objective criteria from international guidance (i.e., related to numeric criteria). The AI component of the software mimics the subjective visual inspection of data usually performed by technicians. Per session it provides an overall session quality grade according to ATS/ERS 2019 Spirometry Technical Statement (A-F) and calculates the best trial (spirometry trace) which should be considered for diagnostic interpretation. The model was trained based on spirometry measurements from the National Health and Nutritional Examination Survey (NHANES 2011-12) (12) and validated in clinical trial settings (14).

The spirometry interpretation component is a decision support software focusing on the diagnostic interpretation of spirometry sessions. It provides support in pattern interpretation according to international interpretation guidelines (i.e. normal, obstructive and/or restrictive) (15) and a disease probability. The disease probability model was originally trained to distinguish eight commonest categories (7 diseases + normal lung function) detectable with full pulmonary function testing (13). For use in primary care settings, the model has been adapted to identify six categories – Asthma, COPD, interstitial lung disease (ILD), normal lung function, other obstructive disease (such as cystic fibrosis and bronchiectasis), and other unidentifiable disease – solely based on spirometry using the same training set. The rationale for reducing the number of categories to identify is based on the reduced lung function dataset available when working in a primary care environment compared to a hospital-based pulmonary function testing laboratory. The categories selected are those most commonly found in primary care, with spirometry testing indicated mainly in COPD and Asthma diagnosis.

For peer review only - http://bmjopen.bmj.com/site/about/guidelines.xhtml

When the quality model identifies the data quality of the spirometry session to be poor (i.e., quality of FEV1 and/or FVC is not A or B according to ATS/ERS 2019 guidelines), the following disclaimer is added to the interpretation report: “Data quality is not sufficient. Interpretation above might not represent the patient’s lung function”. Participants will still be asked for a diagnosis and spirometry interpretation despite the bad quality.

Reference Standard

Reference standards for diagnosis for the 50 spirometry traces were defined before commencement of the trial, by two pulmonologists. Primary and secondary care medical records were reviewed independently by two pulmonologists from the Royal Brompton & Harefield Hospitals, United Kingdom. The diagnostic reference standard was attributed to one of six categories (COPD, Asthma, ILD, OBD, Normal, Other) by each pulmonologist. In participants with multiple respiratory diagnoses (e.g. COPD and ILD), the pulmonologist was asked to choose, to the best of their abilities, the predominant category. If consensus could not be reached between the two pulmonologists, these cases and their medical records were reviewed by a third pulmonologist to adjudicate independently. All pulmonologists (SK, WM, NH) were accredited specialists in respiratory medicine with a minimum of eight years as a consultant in the National Health Service, with expertise in the diagnosis and management of COPD and other chronic respiratory diseases. Pulmonologists had no access to the AI software reports, nor had any communication with the software engineer or software company.

Reference standards for the technical and pattern interpretation were completed by a Respiratory Physiologist (KS) with 26 years’ experience. The Respiratory Physiologist was asked to rate the technical quality of the FEV1 and FVC using the categories Acceptable (quality grade A and B ATS/ERS 2019 Guidelines) or Not Acceptable (Quality grade C D E F U)(15). For pattern interpretation of the spirometry, The Respiratory Physiologist was asked to choose from the following four categories: normal, airflow obstruction, possible restriction or non-specific pattern, possible mixed disorder.

All pulmonologists and respiratory physiologist were blinded to AI software reports.

Study Outcomes

The primary endpoint for a primary care clinician participant is the performance of their preferred diagnosis, defined to be the number of correct cases identified expressed as a percentage out of the total number of spirometry records. A correct case is where the participant’s preferred

For peer review only - http://bmjopen.bmj.com/site/about/guidelines.xhtml

diagnosis matches the reference final diagnosis (described above). The secondary endpoints, expressed per participant are listed in Table 1.

Table 1 Secondary endpoints

Secondary endpoints	Definition	Measured	Reference Standard
Quality assessment of the spirometry trace	A correct case is where the participant's quality grade matches the reference quality grade. Units will be percentage of total cases that are correct	Multiple choice categories: Acceptable (Grade A/B) or Not Acceptable (Grades C/D/E/F/U)	Respiratory Physiologist
Quality Assessment self-rated confidence		Visual Analogue Scale (0-10) where is 0 = not confident at all; 10= very confident)	
Pattern interpretation of the trace	A correct case is where the participants' selected pattern matches the reference pattern.<br>Units will be percentage of total cases that are correct	Multiple choice categories: Normal, Airflow obstruction, Possible restriction or non-specific pattern, Possible Mixed Disorder.	Respiratory Physiologist
Pattern interpretation self-rated confidence		Visual Analogue Scale (0-10) where is 0 = not confident at all; 10= very confident)	
Differential diagnostic performance	A correct case is where the preferred or differential diagnosis matches the reference final diagnosis.<br>Units will be percentage of total cases that are correct	Multiple choice categories: Asthma, COPD, interstitial lung disease (ILD), normal lung function, other obstructive disease, and other unidentifiable disease	Pulmonologist
Diagnostic self-rated confidence		Visual Analogue Scale (0-10) where is 0 = not confident at all; 10= very confident)	

Sample size calculation

The sample size calculation was informed by previous feasibility data from 30 primary care practitioners assessing 5 spirometry traces. The preferred diagnosis matched the reference

For peer review only - http://bmjopen.bmj.com/site/about/guidelines.xhtml

diagnosis in a mean (SD) of 55% (19%) of spirometry records. We have assumed the same SD of 19%, which is conservative because more cases will be assessed per practitioner, leading to less variable practitioner rates. As the calculation involves conservative assumptions, the power was set below 90%, to be 85%. With 132 participants (66 per group) this allows for detection of a 10% difference (a mean of 5 extra cases in 50) in the rate of cases correctly identified to match the reference standard (mean 65% versus mean 55%) using an unpaired t-test at the two-sided 5% significance level.

The recruitment target was originally estimated at 156 practitioners to allow for an estimated 15% drop out. However, a preliminary analysis of completion rate of the first 50 recruited participants demonstrated that 29 of the 50 participants scored all 50 spirometry records within the 8-week study period (58% completion). Based on this data, the study statistician advised increasing the recruitment target to 228 to account for 42% non-completion (see Amendment section).

Descriptive statistics will be performed for demographics, baseline characteristics and endpoint data: number and percentage for categorical data and mean and standard deviation or median inter-quartile range for non-normally distributed) continuous data.

Statistical Analysis

Survey responses from participants will be collated in a secured, online database. A Statistical Analysis Plan (SAP) will be developed and statistical analysis will be conducted by the study statistician. The statistician will be blinded to control/intervention group allocation. Descriptive statistics will be presented for demographic, baseline characteristics and endpoint data. Number and percentage will be presented for categorical data. Mean and standard deviation will be presented for normally distributed continuous data while median inter-quartile range will be presented for non-normally distributed continuous data.

The number of participants consented, randomised, and completing the study will be described in a study CONSORT diagram.

Data analysis will be performed on an Intention to Treat basis. Data will be analysed with the statistical software Stata version 17.0. All statistical tests will be 2-sided and significance set at $p < 0.05$.

For peer review only - http://bmjopen.bmj.com/site/about/guidelines.xhtml

Primary endpoint analysis

For each participant, diagnostic performance will be calculated as percentage of the 50 spirometry procedures where the final diagnosis was correctly identified. In each randomised group, the diagnostic performance will be summarised as the mean of the participants' diagnostic performance. A 2-sample unpaired t test will be used to assess the mean difference in correct response between the 2 randomised groups. The assumptions for the 2-sample t test will be tested and where the assumption for equal variances fails then the Welch t test will be used.

Randomisation will be stratified according to job role (General Practitioner: Yes or No) and Accreditation from Association for Respiratory Technology and Physiology (Performance Only, Interpretation Only, Performance and Interpretation, No accreditation), therefore an analysis of covariance will be used to compare the intervention and control groups by adjusting for the main effects of the stratified variables. To internally validate the estimate of the difference between the 2 groups nonparametric bootstrap estimation of difference will be done by taking 100 from the analysis of covariance model.

Secondary endpoint analysis

For continuous variables, the secondary endpoints will be compared between the control and intervention groups using the same approach as the primary endpoint.

Sub-group analysis

Subgroup analysis will be performed on the 20 spirometry traces where the confirmed diagnosis (reference standard) was COPD, as spirometry in primary care is most relevant to COPD diagnosis.

Missing data

Only participants who complete at least 70% of the spirometry cases will be included in the primary analysis. We will compare the baseline characteristics and demographics of those who completed < 70% of questions with those who completed ≥ 70% of questions to identify any systematic differences between completers and non-completers.

Data Collection

Data will be collected via the online platform Qualtrics which is general data protection regulations (GDPR) compliant. Further information is provided at https://www.qualtrics.com/uk/platform/gdpr/.

For peer review only

For peer review only - http://bmjopen.bmj.com/site/about/guidelines.xhtml

A user license has been obtained by Royal Brompton and Harefield Hospital to use this platform for research purposes. There will be one secure user log in for one member of the study team who will be unblinded. All other members of the research team will be blinded.

Data Management

All data will be handled in accordance with the Data Protection Act (2018), NHS Caldecott Principles, The UK Policy Framework for Health and Social Care Research, and the condition of the REC approval. The online platform (Qualtrics) will be used to collect data responses from each participant reviewing the spirometry traces. Qualtrics itself will act as a database and data will also be downloaded to an excel file/csv file/database) for back up and analysis. Database access will be strictly restricted through user-specific passwords to the authorised research team members. All returned data (Microsoft Forms and Excel spreadsheets) will be held on the H drive (Harefield Hospital) by the clinical team (CI and study team) and will be password protected and only accessible by the direct study team.

No participant identifiable data, beyond basic demographics, will be transferred and all participants will have a participant identification number. Data will be downloaded typically within one week of data collection on Qualtrics by members of the research team. The research team will undertake appropriate reviews of the entered data where appropriate for the purpose of data cleaning and will request amendments as required.

At the end of the study, the CI or their assigned delegate will review all the data for each participant to verify that all the data are complete and correct. At this point, all data can be formally locked for analysis.

Patient and Public Involvement

Patient representatives are included in the trial management group and will attend the quarterly meetings across the duration of the trial. The Deputy Head of Research and Innovation for Asthma + Lung UK (the largest UK Lung Health charity) is also part of the trial management group. Our research team includes two costed co-applicants who will co-lead the PPI elements of the project.

PPI co-applicant responsibilities include: setting and refining overall PPI strategy as the project progresses, reporting on PPI activities to the research management group, evaluation, monitoring

For peer review only - http://bmjopen.bmj.com/site/about/guidelines.xhtml

and reporting of PPI e.g. using a PPI impact log , communicating with our PPI collaborators (Asthma+ Lung UK) as well end-users (GP federations / clinical commissioning groups) on the project status, synthesising results and conclusions of PPI activities and disseminating feedback, writing up PPI sections with public contributors for ethics applications, patient-facing documents and project reports.

Safety Reporting

The procedure proposed for this efficacy study will not affect the usual standard of care for participants or patients. The datasets will comprise spirometry records previously collected as part of clinical spirometry pathways in primary care. As such, this retrospective analysis, which will not involve delivery of an intervention nor a change in patient's usual clinical care, is unlikely to produce direct risk for participants.

The main study risks involve patient confidentiality, information governance and avoiding bias. To address this, all data analysed by parties outside the direct clinical team will be de-identified. Spirometry records will be anonymised and provided to clinicians on an online platform (Qualtrics) compliant with GDPR.

The research team (except the platform administrator) and trial statistician will be blinded to participants' group allocation until the completion of data analysis for the primary and secondary outcomes.

Protocol amendments

Any changes to the study protocol outlined in this paper will be approved by Health Research Authority (HRA) and study Sponsor Royal Brompton and Harefield Hospitals (RBHH), Guy's and St Thomas' NHS Foundation Trust (GSTFT). Two non-substantial amendments have been submitted and approved by HRA for this study: 1. Addition of a link to the study platform in the participant information; 2. An increase in target recruitment number (detailed in Methods section).

For peer review only - http://bmjopen.bmj.com/site/about/guidelines.xhtml

Ethics and Dissemination

Ethical Approval

This study has been reviewed and given favourable opinion by Health Research Authority Wales (Reference: 22/HRA/5023) and registered on Clinical trials.gov (Reference: NCT05933694).

Dissemination

To maximise the audience for dissemination of our findings, results from the study will be disseminated by presentations at relevant scientific meetings and conferences as well as by high impact peer-reviewed publications. We will also disseminate via presentations and newsletters to participants and key stakeholders, including Asthma + Lung UK who have been collaborators on this grant. The results will be shared with local and national primary and secondary care partners and networks, including British Thoracic Society, Primary Care Respiratory Society and International Primary Care Respiratory Group. A summary report will be shared on the Royal Brompton and Harefield website research pages.

Discussion

Spirometry is a key investigation for supporting diagnosis and disease monitoring in chronic respiratory disease. There is a clear need to improve the access, quality and interpretation of spirometry in primary care and other non-respiratory community settings which have many historic challenges, further negatively impacted by the COVID pandemic (8). AI decision-support software has shown potential in improving quality assessment and interpretation of spirometry and this study will evaluate its effectiveness in interpretation of real-world spirometry results assessed by clinicians working in primary care in the UK.

This study is the first randomised controlled efficacy study with an intervention designed to improve the performance of primary care clinicians in the interpretation of spirometry using AI and an important first step for clinicians to make informed choices about its use. It will directly test the effect of an AI-powered spirometry interpretation software in primary care using real-world traces reflective of the results reviewed in practice by clinicians.

We will advertise the trial and recruit nationally to maximise participation and variation in participant sample.

For peer review only - http://bmjopen.bmj.com/site/about/guidelines.xhtml

Funding statement: The study is being funded by National Institute for Health Research (NIHR) through an AI Award in Health and Care (Phase 3- Application: Grant number AI_AWARD02204). The study is also supported by the NIHR Leicester Biomedical Research Centre – Respiratory theme. ST is supported by the National Institute for Health Research ARC North Thames. RR is supported by the NIHR Oxford Biomedical Research Centre – Respiratory. RAE is supported by an NIHR Clinical Scientist fellowship. The views expressed in this publication are those of the authors and not necessarily those of the National Institute for Health Research or the Department of Health and Social Care.

Competing interests: WM is Honorary President of the Association for Respiratory Technology and Physiology (ARTP). MT is CEO of ArtiQ – a company that produces artificial intelligence enabled lung function support software. JM and KVO are employees of ArtiQ.

Authors' contributions: WDC-M and MT conceived the wider research plan. WDC-M, MT, RAE, RR, SSK, SJCT, ATP, NH, NS were co-applicants for the wider grant. All authors developed the design and plan for this study. WB, WDC-M, GD completed the statistical analysis plan which was approved by all authors and ATP provided statistical expertise for the wider grant. MT and ArtiQ team developed the intervention. NS, IJ and NSH led the patient and public involvement. APS and GE set up the study on the Qualtrics platform. GD, EEE and WDC-M drafted the initial manuscript. All authors (GD, EEE, GE, MT, RAE, RR, KPS, KVO, APS, DAS, ATP, JH, SJCT, NSH, SSK, IJ, NS, WB and WD-CM) contributed to the development of the study protocol and reviewed, commented and approved the manuscript.

Acknowledgements: We thank our patient and public involvement members for their contribution to the overall grant application and involvement in trail management meetings. We acknowledge Asthma + Lung UK for their contribution and support with this work.

References

1. Global burden of chronic respiratory diseases and risk factors, 1990-2019: an update from the Global Burden of Disease Study 2019. EClinicalMedicine. 2023;59:101936.
2. IHME G. Global Burden of Disease Study 2019 (GBD 2019) results. Institute for Health Metrics and Evaluation Seattle, United States; 2016.
3. Jones RC, Price D, Ryan D, Sims EJ, von Ziegenweidt J, Mascarenhas L, et al. Opportunities to diagnose chronic obstructive pulmonary disease in routine care in the UK: a retrospective study of a clinical cohort. Lancet Respir Med. 2014;2(4):267-76.

For peer review only - http://bmjopen.bmj.com/site/about/guidelines.xhtml

4. Collard HR, Loyd JE, King TE, Jr., Lancaster LH. Current diagnosis and management of idiopathic pulmonary fibrosis: a survey of academic physicians. Respir Med. 2007;101(9):2011-6.
5. Heffler E, Crimi C, Mancuso S, Campisi R, Puggioni F, Brussino L, et al. Misdiagnosis of asthma and COPD and underuse of spirometry in primary care unselected patients. Respir Med. 2018;142:48-52.
6. NICE. Chronic obstructive pulmonary disease in over 16s: diagnosis and management 2018 [Available from: https://www.nice.org.uk/guidance/ng115.
7. NICE. Asthma: diagnosis, monitoring and chronic asthma management. 2017 [Available from: https://www.nice.org.uk/guidance/ng80.
8. Doe G, Taylor SJ, Topalovic M, Russell R, Evans RA, Maes J, et al. Spirometry services in England post-pandemic and the potential role of AI support software: a qualitative study of challenges and opportunities. British Journal of General Practice. 2023;73(737):e915-e23.
9. van de Hei SJ, Flokstra-de Blok BMJ, Baretta HJ, Doornewaard NE, van der Molen T, Patberg KW, et al. Quality of spirometry and related diagnosis in primary care with a focus on clinical use. NPJ Prim Care Respir Med. 2020;30(1):22.
10. Hegewald MJ, Gallo HM, Wilson EL. Accuracy and Quality of Spirometry in Primary Care Offices. Ann Am Thorac Soc. 2016;13(12):2119-24.
11. Societies FoIR. The global impact of respiratory disease. Third Edition. European Respiratory Society. 2021 2021.
12. Das N, Verstraete K, Stanojevic S, Topalovic M, Aerts J-M, Janssens W. Deep-learning algorithm helps to standardise ATS/ERS spirometric acceptability and usability criteria. European Respiratory Journal. 2020;56(6):2000603.
13. Topalovic M, Das N, Burgel PR, Daenen M, Derom E, Haenebalcke C, et al. Artificial intelligence outperforms pulmonologists in the interpretation of pulmonary function tests. Eur Respir J. 2019;53(4).
14. Desbordes P, Topole E, Biondaro S, Montagna I, Corre S, Topalovic M. AI Over-reading Based on ATS/ERS 2019 Criteria Is a Reliable Option for Instant Spirometry Quality Control in Clinical Trials. B70 INHALE THE FUTURE: NEW FINDINGS IN PULMONARY FUNCTION. p. A4064-A.
15. Graham BL, Steenbruggen I, Miller MR, Barjaktarevic IZ, Cooper BG, Hall GL, et al. Standardization of Spirometry 2019 Update. An Official American Thoracic Society and European Respiratory Society Technical Statement. American Journal of Respiratory and Critical Care Medicine. 2019;200(8):e70-e88.

For peer review only

For peer review only - http://bmjopen.bmj.com/site/about/guidelines.xhtml

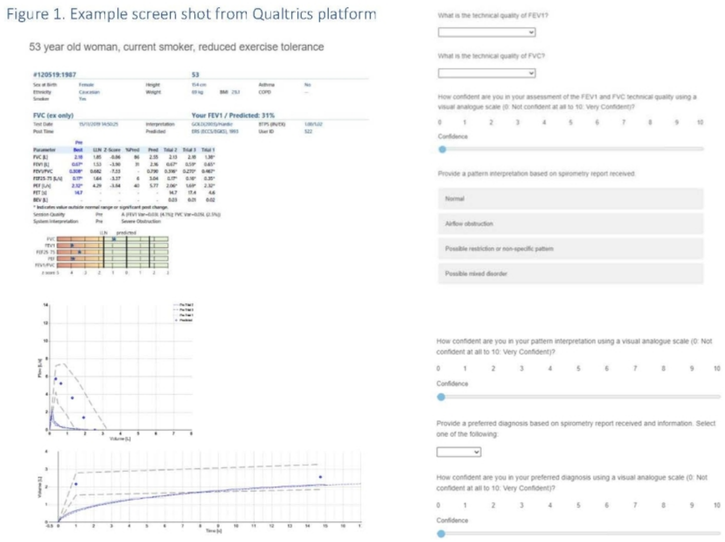

Figure 1. Example spirometry case and questions for primary care clinicians

210x297mm (200 x 200 DPI)

Figure 2. Study schedule

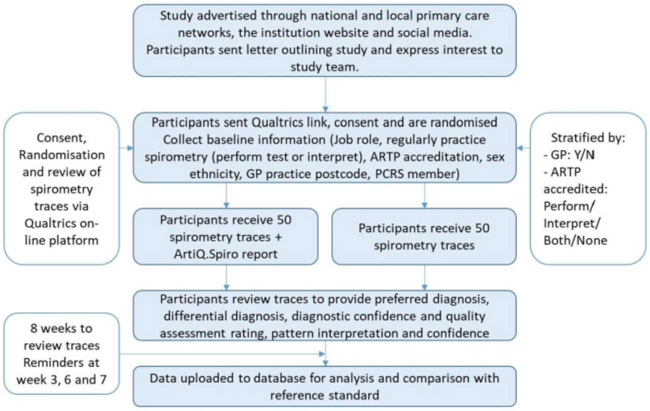

Figure 2. Study Schedule

210x297mm (210 x 210 DPI)

Supplement_Comparing performance of primary care clinicians in the interpretation of SPIROmetry with or without Artificial Intelligence Decision support software (SPIRO-AID): a protocol for a randomised controlled trial.

Supplement

Clinician letter (participant information)

Royal Brompton and Harefield

NHS Guy's and St Thomas' NHS Foundation Trust

IRAS ID: 323361

Invitation to participate in research study: A randomized controlled trial comparing diagnostic performance of primary care clinicians in the interpretation of **SPIRO**metry with or without **A**rtificial **I**ntelligence **D**ecision support software (SPIRO-AID)

Dear Clinician,

We would like to invite you to take part in our research study. This study aims to evaluate whether an artificial intelligence decision support software (ArtiQ. Spiro) improves the diagnostic accuracy of spirometry interpreted by primary care clinicians, as measured by Clinician Diagnostic Accuracy (*vs* Reference Standard). You are being invited to take part because you are a clinician working in primary care who refers patients for spirometry and/or receives spirometry results for review.

The study is being conducted by Harefield Hospital, part of Guy's and St Thomas's NHS Foundation Trust (GSTFT), in partnership with ArtiQ, a company specialising in respiratory medicine and Artificial Intelligence (AI). The Chief Investigator (CI) is Prof William Man, Professor and Honorary Consultant Respiratory Physician. The study is being funded by National Institute for Health Research (NIHR) through an AI Award in Health and Care (Phase 3- Application: Grant number AI_AWARD02204). The lead organisation for the wider research project is Guy's and St Thomas's NHS Foundation Trust. The study is also supported by the NIHR Leicester Biomedical Research Centre - Respiratory.

Please take time to read the following information carefully. Please discuss it with others, including your colleagues if you wish. Please ask us if there is anything that is not clear.

Purpose of the Study: The NHS Long Term Plan (LTP) has identified quality assured spirometry as a clinical priority through investment in training more community staff to perform and interpret spirometry more accurately. However, this process takes time and disagreement over spirometry interpretation can still occur between trained staff. The purpose of this study is to evaluate whether an AI decision support software improves the diagnostic accuracy and quality assessment of spirometry by primary care clinicians.

Taking part: If you are happy to take part in this research please click here where you will be provided with a link to an on-line platform (Qualtrics) to assess your eligibility in the study and to complete a Consent form. You will then be asked about your job role and any previous experience with spirometry, which will help the study team to randomise participants into each arm of the study. Some participants will be randomised to receive

For peer review only - http://bmjopen.bmj.com/site/about/guidelines.xhtml

Supplement_Comparing performance of primary care clinicians in the interpretation of SPIROmetry with or without Artificial Intelligence Decision support software (SPIRO-AID): a protocol for a randomised controlled trial.

50 spirometry traces for review in the usual format that they would be received. Some participants will be randomised to receive 50 spirometry traces plus an AI report (ArtiQ.Spiro). The 50 spirometry traces will be from a de-identified retrospective dataset. We anticipate that reviewing the traces will take approximately one hour of your time.

Expenses and payments: Participants will be offered reimbursement (£150) for their time. Reimbursement details are provided via the Qualtrics study platform after you have completed review of the 50 traces.

Risks and benefits of taking part: The procedure proposed for this study does not affect the usual standard of care for participants. The datasets will comprise spirometry data previously collected as part of clinical spirometry pathways in primary care. As such, this retrospective data analysis, which does not involve delivery of an intervention nor a change in patient's usual clinical care, is unlikely to produce direct risk for participants.

This study will help us to understand if the addition of AI improves diagnostic accuracy and quality assessment of spirometry, informing design of spirometry pathways for the future.

Study period: The study period will be six months. When this period is over, the results will be analysed and written up for dissemination. The results will be presented at scientific meetings and will be published in medical journals. A dissemination event may be arranged and we will invite participants to attend.

Confidentiality: All information which is collected about you during the course of the research will be kept strictly confidential. Royal Brompton and Harefield Hospitals, part of Guy's and St Thomas NHS Foundation Trust is the sponsor for this study. The sponsor will act as the data controller. This means that it is responsible for looking after your information and using it properly. A web based electronic platform (Qualtrics) will be used to collect data responses from each participant reviewing the spirometry traces.

How will we use information about you?

We will need to use information from you for this research project. This information will include your name and contact details. People will use this information to do the research or to check your records to make sure that the research is being done properly.

People who do not need to know who you are will not be able to see your name or contact details. Your data will have a code number instead. We will keep all information about you safe and secure. Database access will be strictly restricted through user-specific passwords to the authorised research team members. At the end of the study, the CI or their assigned delegate will review all the data for each participant to verify that all the data are complete and correct. At this point, all data can be formally locked for analysis.

For peer review only - http://bmjopen.bmj.com/site/about/guidelines.xhtml

Once we have finished the study, we will keep some of the data so we can check the results. We will write our reports in a way that no-one can work out that you took part in the study.

What are your choices about how your information is used?
You can stop being part of the study at any time, without giving a reason, but we will keep information about you that we already have.
We need to manage your records in specific ways for the research to be reliable. This means that we won't be able to let you see or change the data we hold about you.

Where can you find out more about how your information is used?
You can find out more about how we use your information

- at www.hra.nhs.uk/information-about-patients/
- by asking one of the research team
- by sending an email to ig@rbht.nhs.uk, or
- by ringing us on 0207 352 8121 ext. 2610.

Additional information on the use of patient data in research in line with the General Data Protection Regulation (GDPR) is also provided here.

If you have a concern about any aspect of this study you should ask to speak with the researchers who will do their best to answer your questions [Insert local contact number]. If you remain unhappy and wish to complain formally, you can do this. Details can be obtained from the Royal Brompton and Harefield Hospitals (RBHH) Research Office *via* Email: (Insert)

Further Information and Contact Details:
If you require any further information, please do not hesitate to contact us on the details below.

Study email: Insert

Royal Brompton and Harefield Hospital Research Team
Contact [Insert TBC]

Gillian Doe (Research Programme Manager)
Respiratory BRC, Leicester **Tel:** 0116 258 3370 **Email:** ged6@leicester.ac.uk

Yours faithfully,

[Insert signature]
Professor Will Man (CI)

For peer review only - http://bmjopen.bmj.com/site/about/guidelines.xhtml

Supplement_Comparing performance of primary care clinicians in the interpretation of SPIROmetry with or without Artificial Intelligence Decision support software (SPIRO-AID): a protocol for a randomised controlled trial.

Consent form on Study platform

Q15

Please initial each box if you are in agreement

	Initials
I confirm that I have read and understand the information in the Clinician invitation letter dated 07/03/2023 (Version 1.2) for the above study and have had the opportunity to consider the information, ask questions and have these answered satisfactorily.	
I understand that my participation is voluntary and that I am free to withdraw at any time, without giving any reason, without medical care or legal rights being affected.	
I understand that data collected during the study may be looked at by responsible individuals from the study Sponsor or from regulatory authorities where it is relevant to my taking part in research. I give permission to these individuals to have access this data.	
I agree with the publication of the results of this study in a medical journal (all data will be published anonymously).	
I agree to take part in the above study.	

Q16

Name of Participant

Q17

Date

${date://CurrentDate/FL}

Q21

Type your full name below in lieu of a signature

Supplement_Comparing performance of primary care clinicians in the interpretation of SPIROmetry with or without Artificial Intelligence Decision support software (SPIRO-AID): a protocol for a randomised controlled trial.

Example AI-decision support software report

ARTIQ

Session ID: 120519_1987
Analysed on: 2023-03-23 14:50:25 UTC±00:00

Session Evaluation

FEV1 (Grade A) Acceptable
FVC (Grade B) Acceptable

Best Trial Results
Best Trial: 1
FEV1: 0.7 L
FVC: 2.19 L

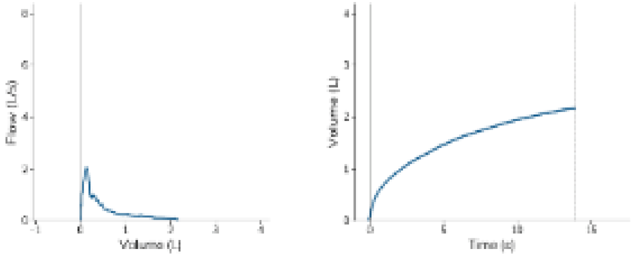

Trial Overview

	FEV1		FVC	
	Acceptable	Usable	Acceptable	Usable
Trial 1	Yes	-	Yes	-
Trial 2	Yes	-	Yes	-
Trial 3	Yes	-	No	Yes

Trial Evaluation Details

TRIAL 1
FEV1 Acceptable
FVC Acceptable

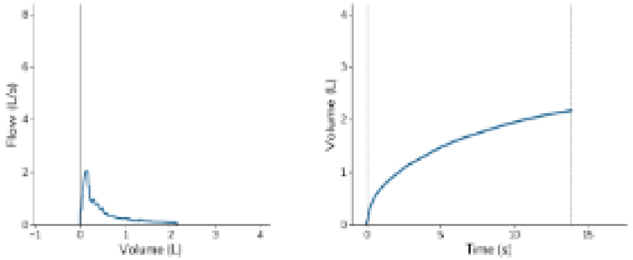

No artefact on MEFVC

BEV Criteria: True
EOFE Criteria: True
Plateau Criteria: False
(39.35 mL change in last second)
tFE > 15s: False
FVC Repeatable: True
(30.34 mL lower than max acceptable FVC)
FIVC-FVC Criteria: None

Warnings
No second inspiration

Page **5** of **7**

Supplement_Comparing performance of primary care clinicians in the interpretation of SPIROmetry with or without Artificial Intelligence Decision support software (SPIRO-AID): a protocol for a randomised controlled trial.

TRIAL 2

FEV1 Acceptable

FVC Acceptable

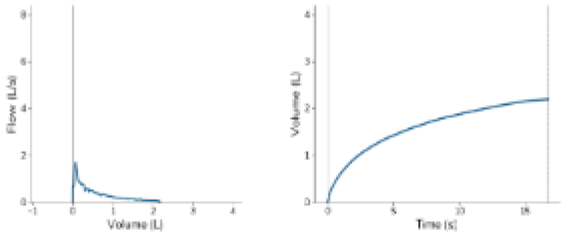

BEV Criteria: True

EOFE Criteria: True

Plateau Criteria: True

(19.9 mL change in last second)

tFE > 15s: True

FVC Repeatable: True

(0.0 mL lower than max acceptable FVC)

FIVC-FVC Criteria: None

No artefact on MEFVC

Warnings

No second inspiration

TRIAL 3

FEV1 Acceptable

FVC Usable (Failure of EOFE criteria)

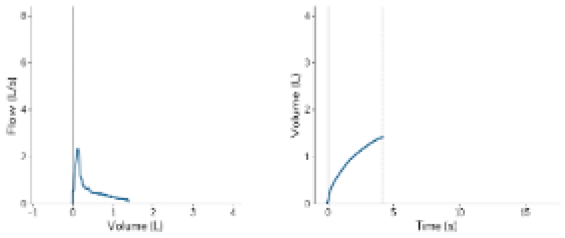

BEV Criteria: True

EOFE Criteria: False

Plateau Criteria: False

(143.41 mL change in last second)

tFE > 15s: False

FVC Repeatable: False

(780.41 mL lower than max acceptable FVC)

FIVC-FVC Criteria: None

No artefact on MEFVC

Warnings

No second inspiration

*Note no second inspiration present on these curves as this is a retrospective dataset where second inspiration was not captured

For peer review only - http://bmjopen.bmj.com/site/about/guidelines.xhtml

Supplement_Comparing performance of primary care clinicians in the interpretation of SPIROmetry with or without Artificial Intelligence Decision support software (SPIRO-AID): a protocol for a randomised controlled trial.

ARTIQ

Analyzed: 2023-04-18 23:33 UTC±00:00

Report ID: _120519_1987

Age: 53 **Gender:** Female **Current Smoker:** Yes **Pack-Years:** N/A

Interpretation of lung function tests

Spirometry demonstrates an obstructive pattern, but a restrictive component is not excluded.

Disease probability

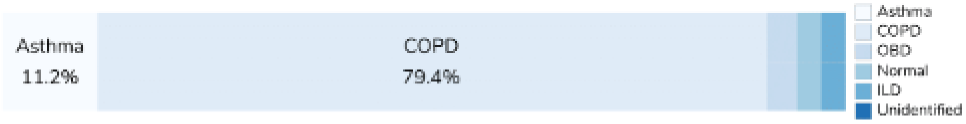

Conclusions and suggestions

Highest disease probability based on lung function: **COPD**

Consider referring to specialist and/or further testing as appropriate:
Careful auscultation on all lung areas for presence of rhonchi. Repeat spirometry with bronchodilator test. Perform screening laboratory (including eosinophilia). Perform X-ray of the thorax. Consider bronchodilator therapy.

Warnings

Probability of disease presence may not be accurate due to missing correct information of pack-years.

Legend

COPD	Chronic Obstructive Pulmonary Disease
OBD	Other Obstructive Diseases (including: cystic fibrosis, bronchiectasis, bronchiolitis)
Normal	Normal lung function
ILD	Interstitial lung disease (including: idiopathic pulmonary fibrosis, nonspecific interstitial pneumonitis, sarcoidosis)
Unidentified	Unidentified (including: neuromuscular disease, pulmonary vascular disease, thoracic deformity, pleural disease)

This report is approved for clinical use in the EU.
Automatically generated by ArtiQ.PFT VERSION NOT SET • Manufactured by ArtiQ NV • Leuven, Belgium

info@ArtiQ.eu
www.ArtiQ.eu

SPIRIT

STANDARD PROTOCOL ITEMS: RECOMMENDATIONS FOR INTERVENTIONAL TRIALS

SPIRIT 2013 Checklist: Recommended items to address in a clinical trial protocol and related documents*

Section/item	ItemNo	Description	Page no.
**Administrative information**			
Title	1	Descriptive title identifying the study design, population, interventions, and, if applicable, trial acronym	1
Trial registration	2a	Trial identifier and registry name. If not yet registered, name of intended registry	2<br>Clinical trials.gov (Reference: NCT05933694).
	2b	All items from the World Health Organization Trial Registration Data Set	
Protocol version	3	Date and version identifier	Version 1 for Publication
Funding	4	Sources and types of financial, material, and other support	18<br>Funded by National Institute for Health Research (NIHR) through an AI Award in Health and Care (Phase 3-Application: Grant number AI_AWARD02204
Roles and responsibilities	5a	Names, affiliations, and roles of protocol contributors	1
	5b	Name and contact information for the trial sponsor	4<br>Royal Brompton and Harefield Hospitals (RBHH), Guy's and St Thomas' NHS Foundation Trust (GSTFT)

For peer review only

	5c	Role of study sponsor and funders, if any, in study design; collection, management, analysis, and interpretation of data; writing of the report; and the decision to submit the report for publication, including whether they will have ultimate authority over any of these activities	18
	5d	Composition, roles, and responsibilities of the coordinating centre, steering committee, endpoint adjudication committee, data management team, and other individuals or groups overseeing the trial, if applicable (see Item 21a for data monitoring committee)	18
**Introduction**			
Background and rationale	6a	Description of research question and justification for undertaking the trial, including summary of relevant studies (published and unpublished) examining benefits and harms for each intervention	3 and 4
	6b	Explanation for choice of comparators	
Objectives	7	Specific objectives or hypotheses	4
Trial design	8	Description of trial design including type of trial (eg, parallel group, crossover, factorial, single group), allocation ratio, and framework (eg, superiority, equivalence, noninferiority, exploratory)	4
**Methods: Participants, interventions, and outcomes**			

For peer review only

Study setting	9	Description of study settings (eg, community clinic, academic hospital) and list of countries where data will be collected. Reference to where list of study sites can be obtained	6
Eligibility criteria	10	Inclusion and exclusion criteria for participants. If applicable, eligibility criteria for study centres and individuals who will perform the interventions (eg, surgeons, psychotherapists)	5
Interventions	11a	Interventions for each group with sufficient detail to allow replication, including how and when they will be administered	10 and 11
	11b	Criteria for discontinuing or modifying allocated interventions for a given trial participant (eg, drug dose change in response to harms, participant request, or improving/worsening disease)	
	11c	Strategies to improve adherence to intervention protocols, and any procedures for monitoring adherence (eg, drug tablet return, laboratory tests)	
	11d	Relevant concomitant care and interventions that are permitted or prohibited during the trial	n/a
Outcomes	12	Primary, secondary, and other outcomes, including the specific measurement variable (eg, systolic blood pressure), analysis metric (eg, change from baseline, final value, time to event), method of aggregation (eg, median, proportion), and time point for each outcome. Explanation of the clinical relevance of chosen efficacy and harm outcomes is strongly recommended	12,13

Participant timeline	13	Time schedule of enrolment, interventions (including any run-ins and washouts), assessments, and visits for participants. A schematic diagram is highly recommended (see Figure)	10<br>Figure 2 – study schedule
Sample size	14	Estimated number of participants needed to achieve study objectives and how it was determined, including clinical and statistical assumptions supporting any sample size calculations	13 and 14
Recruitment	15	Strategies for achieving adequate participant enrolment to reach target sample size	14
**Methods: Assignment of interventions (for controlled trials)**			
Allocation:			
Sequence generation	16a	Method of generating the allocation sequence (eg, computer-generated random numbers), and list of any factors for stratification. To reduce predictability of a random sequence, details of any planned restriction (eg, blocking) should be provided in a separate document that is unavailable to those who enrol participants or assign interventions	5
Allocation concealment mechanism	16b	Mechanism of implementing the allocation sequence (eg, central telephone; sequentially numbered, opaque, sealed envelopes), describing any steps to conceal the sequence until interventions are assigned	
Implementation	16c	Who will generate the allocation sequence, who will enrol participants, and who will assign participants to interventions	5 and 6

For peer review only - http://bmjopen.bmj.com/site/about/guidelines.xhtml

Blinding (masking)	17a	Who will be blinded after assignment to interventions (eg, trial participants, care providers, outcome assessors, data analysts), and how	6, 12, 14 and 15
	17b	If blinded, circumstances under which unblinding is permissible, and procedure for revealing a participant's allocated intervention during the trial	
**Methods: Data collection, management, and analysis**			
Data collection methods	18a	Plans for assessment and collection of outcome, baseline, and other trial data, including any related processes to promote data quality (eg, duplicate measurements, training of assessors) and a description of study instruments (eg, questionnaires, laboratory tests) along with their reliability and validity, if known. Reference to where data collection forms can be found, if not in the protocol	15 and 16
	18b	Plans to promote participant retention and complete follow-up, including list of any outcome data to be collected for participants who discontinue or deviate from intervention protocols	9 and 10<br>Figure 2
Data management	19	Plans for data entry, coding, security, and storage, including any related processes to promote data quality (eg, double data entry; range checks for data values). Reference to where details of data management procedures can be found, if not in the protocol	15 and 16

Statistical methods	20a	Statistical methods for analysing primary and secondary outcomes. Reference to where other details of the statistical analysis plan can be found, if not in the protocol	13 and 14
	20b	Methods for any additional analyses (eg, subgroup and adjusted analyses)	15
	20c	Definition of analysis population relating to protocol non-adherence (eg, as randomised analysis), and any statistical methods to handle missing data (eg, multiple imputation)	15
**Methods: Monitoring**			
Data monitoring	21a	Composition of data monitoring committee (DMC); summary of its role and reporting structure; statement of whether it is independent from the sponsor and competing interests; and reference to where further details about its charter can be found, if not in the protocol. Alternatively, an explanation of why a DMC is not needed	15 and 16 – data management.<br>No DMC<br>Data will be monitored, checked and cleaned by the study team.
	21b	Description of any interim analyses and stopping guidelines, including who will have access to these interim results and make the final decision to terminate the trial	15
Harms	22	Plans for collecting, assessing, reporting, and managing solicited and spontaneously reported adverse events and other unintended effects of trial interventions or trial conduct	16 and 17

For peer review only - http://bmjopen.bmj.com/site/about/guidelines.xhtml

Auditing	23	Frequency and procedures for auditing trial conduct, if any, and whether the process will be independent from investigators and the sponsor	The Sponsor operates a risk based monitoring and audit programme, to which this study will be subject.
**Ethics and dissemination**			
Research ethics approval	24	Plans for seeking research ethics committee/institutional review board (REC/IRB) approval	2<br>This study has been reviewed and given favourable opinion by Health Research Authority Wales (Reference: 22/HRA/5023).
Protocol amendments	25	Plans for communicating important protocol modifications (eg, changes to eligibility criteria, outcomes, analyses) to relevant parties (eg, investigators, REC/IRBs, trial participants, trial registries, journals, regulators)	17
Consent or assent	26a	Who will obtain informed consent or assent from potential trial participants or authorised surrogates, and how (see Item 32)	5 and 6
	26b	Additional consent provisions for collection and use of participant data and biological specimens in ancillary studies, if applicable	n/a
Confidentiality	27	How personal information about potential and enrolled participants will be collected, shared, and maintained in order to protect confidentiality before, during, and after the trial	Supplement<br>- Clinician letter (participant information sheet)
Declaration of interests	28	Financial and other competing interests for principal investigators for the overall trial and each study site	18

For peer review only - http://bmjopen.bmj.com/site/about/guidelines.xhtml

Access to data	29	Statement of who will have access to the final trial dataset, and disclosure of contractual agreements that limit such access for investigators	
Ancillary and post-trial care	30	Provisions, if any, for ancillary and post-trial care, and for compensation to those who suffer harm from trial participation	Participation is considered to be very low risk and does not involve patients.
Dissemination policy	31a	Plans for investigators and sponsor to communicate trial results to participants, healthcare professionals, the public, and other relevant groups (eg, via publication, reporting in results databases, or other data sharing arrangements), including any publication restrictions	17
	31b	Authorship eligibility guidelines and any intended use of professional writers	
	31c	Plans, if any, for granting public access to the full protocol, participant-level dataset, and statistical code	
**Appendices**			
Informed consent materials	32	Model consent form and other related documentation given to participants and authorised surrogates	Supplement
Biological specimens	33	Plans for collection, laboratory evaluation, and storage of biological specimens for genetic or molecular analysis in the current trial and for future use in ancillary studies, if applicable	n/a

*It is strongly recommended that this checklist be read in conjunction with the SPIRIT 2013 Explanation & Elaboration for important clarification on the items. Amendments to the protocol should be tracked and dated. The SPIRIT checklist is copyrighted by the SPIRIT Group under the Creative Commons "Attribution-NonCommercial-NoDerivs 3.0 Unported" license.

For peer review only

BMJ Open

Comparing performance of primary care clinicians in the interpretation of SPIROmetry with or without Artificial Intelligence Decision support software (SPIRO-AID): a protocol for a randomised controlled trial.

Journal:	*BMJ Open*
Manuscript ID	bmjopen-2024-086736.R2
Article Type:	Protocol
Date Submitted by the Author:	28-May-2024
Complete List of Authors:	Doe, Gillian; University of Leicester, NIHR Biomedical Research Centre<br>El-Emir, E; Guy's and St Thomas' NHS Foundation Trust<br>Edwards, G; Guy's and St Thomas' NHS Foundation Trust<br>Topalovic, Marko; ARTIQ<br>Evans , Rachael; University of Leicester, NIHR Biomedical Research Centre<br>Russell, Richard; King's College London, Kings Centre for Lung Health<br>Sylvester, Karl; Cambridge University Hospitals NHS Foundation Trust, Cambridge Respiratory Physiology<br>Orshoven, K; ARTIQ<br>Sunjaya, A; The George Institute for Global Health; Guy's and St Thomas' Hospitals NHS Trust<br>Scott, D; University of Southampton, Southampton Health Technology Assessments Centre<br>Prevost, A Toby; King's College London, Nightingale-Saunders Clinical Trials and Epidemiology Unit<br>Harvey, J; Guy's and St Thomas' Hospitals NHS Trust<br>Taylor, Stephanie; Queen Mary University of London, Wolfson Institute of Population Health<br>Hopkinson, Nicholas; Imperial College London, National Heart and Lung Insititute<br>Kon, S; Hillingdon Hospitals NHS Foundation Trust, Department of Respiratory Medicine; Guy's and St Thomas' NHS Foundation Trust, Department of Respiratory Medicine<br>Jarrold, I; Asthma and Lung UK<br>Spain, N; PPI partner<br>Banya, W; Guy's and St Thomas' Hospitals NHS Trust<br>Man, William; Guy's and St Thomas' Hospitals NHS Trust; King's College London, King’s Centre for Lung Health
**Primary Subject Heading**:	Respiratory medicine
Secondary Subject Heading:	Diagnostics, General practice / Family practice
Keywords:	RESPIRATORY MEDICINE (see Thoracic Medicine), Primary Care < Primary Health Care, Lung Diseases

SCHOLARONE™
Manuscripts

Title: Comparing performance of primary care clinicians in the interpretation of SPIROmetry with or without Artificial Intelligence Decision support software (SPIRO-AID): a protocol for a randomised controlled trial.

Authors:

Doe G[1], El-Emir E[2], Edwards G[2], Topalovic M[3], Evans RA[1], Russell R[4], [3], Sylvester KP[5], Van Orshoven K[3], Sunjaya AP[2,6], Scott DA[7], Prevost AT[8], Harvey J[2], Taylor SJC[9], Hopkinson NS[10], Kon SS[2,11], Jarrold I[12], Spain N[13], Banya W[2,14] and Man WD-C[2, 4, 10]

Institutions:

1. NIHR Biomedical Research Centre (Respiratory Theme), Department of Respiratory Science, University of Leicester, Leicester, UK
2. Harefield Respiratory Research Group, Royal Brompton & Harefield Hospitals, Guy's and St Thomas' NHS Foundation Trust, UK
3. ARTIQ, Leuven, Belgium
4. King's Centre for Lung Health, Faculty of Life Sciences and Medicine, King's College, London.
5. Royal Papworth Hospital NHS Foundation Trust, UK
6. The George Institute for Global Health, UNSW Sydney, Australia and The George Institute for Global Health, Imperial College London, UK
7. Southampton Health Technology Assessments Centre, University of Southampton, Southampton, UK; Diabetes Research Centre, University of Leicester, Leicester, UK
8. Nightingale-Saunders Clinical Trials and Epidemiology Unit, Cicely Saunders Institute of Palliative Care, Policy and Rehabilitation, Kings College London, UK
9. Wolfson Institute of Population Health, Queen Mary University of London, London, UK
10. National Heart & Lung Institute, Imperial College London, UK
11. Department of Respiratory Medicine, The Hillingdon Hospitals NHS Foundation Trust, UK
12. Asthma + Lung UK
13. Patient and public involvement representative, UK
14. Medical Statistics, Research & Development, Royal Brompton & Harefield Hospitals, Guy's and St Thomas' NHS Foundation Trust, UK

Author for correspondence: Gillian Doe, Leicester NIHR Biomedical Research Centre – Respiratory, Glenfield Hospital, Leicester LE3 9QP

Email: ged6@leicester.ac.uk ORCID ID: 0000-0003-4782-581

For peer review only

Abstract

Introduction: Spirometry is a point-of-care lung function test that helps support the diagnosis and monitoring of chronic lung disease. The quality and interpretation accuracy of spirometry is variable in primary care. This study aims to evaluate whether artificial intelligence (AI) decision support software improves the performance of primary care clinicians in the interpretation of spirometry, against reference standard (expert interpretation)

Methods and Analysis: A parallel, two-group, statistician-blinded, randomised controlled trial of primary care clinicians in the United Kingdom, who refer for, or interpret, spirometry. People with specialist training in respiratory medicine to consultant level were excluded. A minimum target of 228 primary care clinician participants will be randomised with a 1:1 allocation to assess fifty de-identified, real-world patient spirometry sessions through an on-line platform either with (Intervention group) or without (Control group) AI decision support software report. Outcomes will cover primary care clinicians' spirometry interpretation performance including measures of technical quality assessment, spirometry pattern recognition and diagnostic prediction, compared with reference standard. Clinicians' self-rated confidence in spirometry interpretation will also be evaluated. The primary outcome is the proportion of the fifty spirometry sessions where the participant's preferred diagnosis matches the reference diagnosis. Unpaired t-tests and Analysis of Covariance will be used to estimate the difference in primary outcome between intervention and control groups.

Ethics and Dissemination: This study has been reviewed and given favourable opinion by Health Research Authority Wales (Reference: 22/HRA/5023) and registered on Clinical trials.gov (Reference: NCT05933694).

Results will be submitted for publication in peer-reviewed journals, presented at relevant national and international conferences, disseminated through social media, patient and public routes and directly shared with stakeholders.

Trial registration: NCT05933694

For peer review only

For peer review only - http://bmjopen.bmj.com/site/about/guidelines.xhtml

Strengths and Limitations:

- Real-world spirometry traces completed in primary care, irrespective of technical quality, will be used.
- To replicate real-world primary care in the United Kingdom, study participants will not be limited to general practitioners, but will include other members of multidisciplinary team that are expected to perform or/and interpret spirometry in primary care.
- The trial will be advertised and recruited nationally to maximise participation and variation in participant sample.
- The trial statistician will be blinded to group allocation.
- Trial outcomes will include all relevant aspects of spirometry interpretation, including assessment of technical quality, pattern recognition and diagnosis prediction.

Introduction

Chronic respiratory disease has significant negative impact on quality of life and is the third leading cause of death globally, and one of the highest contributors to economic burden for healthcare systems (1). Chronic Obstructive Pulmonary Disease (COPD) alone accounted for 3.3 million deaths worldwide in 2019, increasing 14% over the previous ten years (2). Accurate and timely diagnosis of respiratory disease is key to improving access to treatment and patient outcomes, and long delays to diagnosis for individuals with chronic lung diseases are well documented (3-5).

Spirometry is a simple, point-of-care lung function procedure recommended to support the diagnosis and monitoring of COPD and asthma, the commonest long-term respiratory conditions (6, 7). In many western and low and middle income (LMIC) countries, spirometry will be performed and interpreted in non- specialist respiratory settings. However, there is inequity in spirometry provision in primary care and community settings; historical challenges with quality, skills and competence in interpretation, and funding, have been compounded by the COVID-19 pandemic (8). Spirometry

For peer review only - http://bmjopen.bmj.com/site/about/guidelines.xhtml

performed in primary care and community settings has been shown to not meet international criteria in over 85% of cases (9). A low level of confidence in assessing spirometry quality is also common amongst general practitioners (10). Improving prevention and early diagnosis has been identified as a priority in the Global Impact of Respiratory Disease report (11). In practice, this requires improved equity, access and quality of spirometry provision.

Recent work has shown that Artificial Intelligence (AI)–powered decision support software can provide automated technical quality assessment and interpretation of full lung function tests (12, 13). This software can outperform pulmonologists in diagnostic prediction from lung function tests (13). Similar AI software that interprets spirometry (usually the only lung function test accessible in many healthcare settings) may be particularly helpful in primary care to support clinicians who have generalist roles and less experience in respiratory diagnosis. Qualitative work highlighted that validation and evaluation of the effectiveness of AI software is key in engaging clinicians and commissioners to have confidence in implementation in clinical practice.

The primary objective of this study is to compare the performance of primary care clinicians in the interpretation of 50 real-world spirometry records with or without AI spirometry decision support software reports.

Methods and Analysis

Trial Design and Registration

This is a parallel, two group, randomised controlled, statistician-blinded efficacy trial to evaluate whether AI spirometry decision support software can improve the performance of primary care clinicians in their interpretation of real-world spirometry. Each clinician will be provided with the same clinical dataset of 50 de-identified, real-world patient spirometry records through an online platform (Qualtrics XM), and asked to answer questions about preferred diagnosis (i.e. diagnosis they think is most likely) and differential diagnosis (i.e. second most likely diagnosis) based on spirometry and limited clinical data, the technical quality of spirometry and the spirometry pattern. Questions will also explore clinicians' confidence in their assessments.

For peer review only - http://bmjopen.bmj.com/site/about/guidelines.xhtml

Participants will be allocated at random to receive either spirometry records alone or spirometry records with the addition of the AI software report. The clinical spirometry records will be de-identified (name, date of birth, address, postcode, occupation, GP, medications data removed), by a member of the clinical care team.

Royal Brompton and Harefield Hospitals (RBHH), Guy's and St Thomas' NHS Foundation Trust (GSTFT) will act as study sponsor and the trial has been registered with clinicaltrials.gov (NCT05933694). This study has been reviewed and given favourable opinion by Health Research Authority Wales (Reference: 22/HRA/5023).

Study Population

Potential participants will be clinicians working in primary care involved in spirometry referral and interpretation.

Eligibility Criteria

Eligibility criteria will be: 1) A clinician working in primary care (defined as >50% of job plan in primary care) in the United Kingdom who refers for or performs spirometry (typically GP, practice nurse); 2) Able to access an online study platform in order to review spirometry sessions; 3) Able to provide informed consent via the study platform.

Exclusion criterion will be: 1) Clinicians who have completed specialist training in respiratory medicine and are recognised by the General Medical Council with the right to practise as a consultant in respiratory medicine in the National Health Service.

Eligibility criteria will be used as screening questions on the on-line study platform prior to participants being able to consent to participate.

Participants will be randomised 1:1 to either control (report 50 spirometry records alone) or intervention (report same 50 spirometry records with access to an AI spirometry interpretation report for each spirometry result). Randomisation will be done by the study on-line platform at the point of consent to the study by minimising according to job role (General Practitioner: Yes or No) and spirometry accreditation from the Association for Respiratory Technology and Physiology (Performance Only, Interpretation Only, Performance and Interpretation, No accreditation) and then randomising by a random number generation algorithm performed by the on-line platform.

For peer review only - http://bmjopen.bmj.com/site/about/guidelines.xhtml

Recruitment and Consent

Recruitment to the study will be advertised through national and local primary care networks, professional networks, the institution website and social media will be used. By advertising and recruiting nationally, the recruitment strategy aims to engage with clinicians from diverse backgrounds in terms of the patient population they serve, age, years of clinical experience, job role and ethnicity. We will offer financial reimbursement for all potential participants participating and completing the study, which may attract some who might not be interested in AI or respiratory diagnostics.

Clinicians will be sent a letter outlining the research study with the option for them to express interest in participating. The letter serves as the participant information sheet (see supplementary information) and will be sent via email. Participants who express interest in taking part will be sent a link to the on-line study platform where they will be able to complete the eligibility screening questions and, if applicable, consent form online. The study will also be advertised on the Royal Brompton and Harefield Hospitals Research website with a link available to the on-line study platform. Participants will be able to withdraw without giving any reason, and the number of participants registering but not completing the study will be recorded. Randomisation will be kept concealed until the point of the allocation of the participant to a study arm.

For peer review only

Setting

The study will be conducted via an online platform (Qualtrics XM).

Procedure

Study participants (participating clinicians) will independently assess the same 50 spirometry records, in the same order, through a bespoke designed online platform developed using Qualtrics XM (Seattle, USA). Participants will be blinded to the reference standards. Please see Figure 1 for example. The patient spirometry records were randomly selected from a database comprising 1122 patients undergoing spirometry in primary care and community-based respiratory clinics in Hillingdon borough between 2015-2019. An online randomizer will be used to select the 50 spirometry sessions used in the trial (https://www.randomizer.org/), according to "disease category" so that of the 50 spirometry traces selected, 40% will be from patients with COPD, 20% will have

Normal spirometry, and 10% for each of the four other disease categories (Asthma, ILD, Other obstructive, Other disease/unidentifiable category), to represent a likely distribution of diagnoses in a primary care setting.

For each spirometry record, the primary care clinician participant will answer the following questions on the Qualtrics platform:

- What is the Technical Quality of the spirometry? (a drop-down box containing the options: Acceptable (Quality grade A or B), Not acceptable). Technical quality will be defined according to the ATS/ERS 2019 Spirometry Technical Statement, provided to participants via a link embedded in the on-line platform
    - One response for Forced expiratory volume in one second (FEV_1) and one question for Forced Vital Capacity (FVC)
- How confident are you in your technical quality assessment using a visual analogue scale (0: Not confident at all to 10: Very Confident)?
- Provide a pattern interpretation based on spirometry report received. Options: Normal, Airflow Obstruction, Possible restriction or non-specific pattern, Possible mixed disorder
- How confident are you in your pattern interpretation using a visual analogue scale (0: Not confident at all to 10: Very Confident)?
- Provide a preferred diagnosis based on spirometry report received and information. (a drop-down box containing the options: Asthma, COPD, interstitial lung disease (ILD), normal lung function, other obstructive disease, and other unidentifiable disease): **Preferred Diagnosis**
- Provide a differential diagnosis based on spirometry report received and information. (a drop-down box containing the options: Asthma, COPD, interstitial lung disease (ILD), normal lung function, other obstructive disease, and other unidentifiable disease)**: Differential Diag**nosis. The previously chosen 'preferred diagnosis' is automatically removed from the list of available options.
- How confident are you in your diagnosis using a visual analogue scale (0: Not confident at all to 10: Very Confident)

For peer review only

For peer review only - http://bmjopen.bmj.com/site/about/guidelines.xhtml

Participants will have eight weeks to review the 50 spirometry traces; reminders will be sent at weeks three, six and seven to those who have not completed their assessments. (Figure 2).

Intervention – AI spirometry software

The AI software was developed by ArtiQ (Leuven, Belgium), and provides AI-supported quality assessment and interpretation guidance of spirometry sessions (Supplement). The quality assessment component leverages deep learning methods to perform the subjective assessment of spirometry quality assessment (i.e., related to curve shape), as well as implementing the objective criteria from international guidance (i.e., related to numeric criteria). The AI component of the software mimics the subjective visual inspection of data usually performed by technicians. Per session it provides an overall session quality grade according to ATS/ERS 2019 Spirometry Technical Statement (A-F) and calculates the best trial (spirometry trace) which should be considered for diagnostic interpretation. The model was trained based on spirometry measurements from the National Health and Nutritional Examination Survey (NHANES 2011-12) (12) and validated in clinical trial settings (14).

The spirometry interpretation component is a decision support software focusing on the diagnostic interpretation of spirometry sessions. It provides support in pattern interpretation according to international interpretation guidelines (i.e. normal, obstructive and/or restrictive) (15) and a disease probability. The disease probability model was originally trained to distinguish eight commonest categories (7 diseases + normal lung function) detectable with full pulmonary function testing (13). For use in primary care settings, the model has been adapted to identify six categories – Asthma, COPD, interstitial lung disease (ILD), normal lung function, other obstructive disease (such as cystic fibrosis and bronchiectasis), and other unidentifiable disease – solely based on spirometry using the same training set. The rationale for reducing the number of categories to identify is based on the reduced lung function dataset available when working in a primary care environment compared to a hospital-based pulmonary function testing laboratory. The categories selected are those most commonly found in primary care, with spirometry testing indicated mainly in COPD and Asthma diagnosis.

For peer review only - http://bmjopen.bmj.com/site/about/guidelines.xhtml

For peer review only

When the quality model identifies the data quality of the spirometry session to be poor (i.e., quality of FEV1 and/or FVC is not A or B according to ATS/ERS 2019 guidelines), the following disclaimer is added to the interpretation report: “Data quality is not sufficient. Interpretation above might not represent the patient’s lung function”. Participants will still be asked for a diagnosis and spirometry interpretation despite the bad quality.

Reference Standard

Reference standards for diagnosis for the 50 spirometry traces were defined before commencement of the trial, by two pulmonologists. Primary and secondary care medical records were reviewed independently by two pulmonologists from the Royal Brompton & Harefield Hospitals, United Kingdom. The diagnostic reference standard was attributed to one of six categories (COPD, Asthma, ILD, OBD, Normal, Other) by each pulmonologist. In participants with multiple respiratory diagnoses (e.g. COPD and ILD), the pulmonologist was asked to choose, to the best of their abilities, the predominant category. If consensus could not be reached between the two pulmonologists, these cases and their medical records were reviewed by a third pulmonologist to adjudicate independently. All pulmonologists (SK, WM, NH) were accredited specialists in respiratory medicine with a minimum of eight years as a consultant in the National Health Service, with expertise in the diagnosis and management of COPD and other chronic respiratory diseases. Pulmonologists had no access to the AI software reports, nor had any communication with the software engineer or software company.

Reference standards for the technical and pattern interpretation were completed by a Respiratory Physiologist (KS) with 26 years’ experience. The Respiratory Physiologist was asked to rate the technical quality of the FEV1 and FVC using the categories Acceptable (quality grade A and B ATS/ERS 2019 Guidelines) or Not Acceptable (Quality grade C D E F U)(15). For pattern interpretation of the spirometry, The Respiratory Physiologist was asked to choose from the following four categories: normal, airflow obstruction, possible restriction or non-specific pattern, possible mixed disorder.

All pulmonologists and respiratory physiologist were blinded to AI software reports.

Study Outcomes

The primary endpoint for a primary care clinician participant is the performance of their preferred diagnosis, defined to be the number of correct cases identified expressed as a percentage out of the total number of spirometry records. A correct case is where the participant’s preferred

For peer review only

diagnosis matches the reference final diagnosis (described above). The secondary endpoints, expressed per participant are listed in Table 1.

Table 1 Secondary endpoints

Secondary endpoints	Definition	Measured	Reference Standard
Quality assessment of the spirometry trace	A correct case is where the participant's quality grade matches the reference quality grade. Units will be percentage of total cases that are correct	Multiple choice categories: Acceptable (Grade A/B) or Not Acceptable (Grades C/D/E/F/U)	Respiratory Physiologist
Quality Assessment self-rated confidence		Visual Analogue Scale (0-10) where is 0 = not confident at all; 10= very confident)	
Pattern interpretation of the trace	A correct case is where the participants' selected pattern matches the reference pattern.<br>Units will be percentage of total cases that are correct	Multiple choice categories: Normal, Airflow obstruction, Possible restriction or non-specific pattern, Possible Mixed Disorder.	Respiratory Physiologist
Pattern interpretation self-rated confidence		Visual Analogue Scale (0-10) where is 0 = not confident at all; 10= very confident)	
Differential diagnostic performance	A correct case is where the preferred or differential diagnosis matches the reference final diagnosis.<br>Units will be percentage of total cases that are correct	Multiple choice categories: Asthma, COPD, interstitial lung disease (ILD), normal lung function, other obstructive disease, and other unidentifiable disease	Pulmonologist
Diagnostic self-rated confidence		Visual Analogue Scale (0-10) where is 0 = not confident at all; 10= very confident)	

Sample size calculation

The sample size calculation was informed by previous feasibility data from 30 primary care practitioners assessing 5 spirometry traces. The preferred diagnosis matched the reference

For peer review only - http://bmjopen.bmj.com/site/about/guidelines.xhtml

diagnosis in a mean (SD) of 55% (19%) of spirometry records. We have assumed the same SD of 19%, which is conservative because more cases will be assessed per practitioner, leading to less variable practitioner rates. As the calculation involves conservative assumptions, the power was set below 90%, to be 85%. With 132 participants (66 per group) this allows for detection of a 10% difference (a mean of 5 extra cases in 50) in the rate of cases correctly identified to match the reference standard (mean 65% versus mean 55%) using an unpaired t-test at the two-sided 5% significance level.

The recruitment target was originally estimated at 156 practitioners to allow for an estimated 15% drop out. However, a preliminary analysis of completion rate of the first 50 recruited participants demonstrated that 29 of the 50 participants scored all 50 spirometry records within the 8-week study period (58% completion). Based on this data, the study statistician advised increasing the recruitment target to 228 to account for 42% non-completion (see Amendment section).

Descriptive statistics will be performed for demographics, baseline characteristics and endpoint data: number and percentage for categorical data and mean and standard deviation or median inter-quartile range for non-normally distributed) continuous data.

Statistical Analysis

Survey responses from participants will be collated in a secured, online database. A Statistical Analysis Plan (SAP) will be developed and statistical analysis will be conducted by the study statistician. The statistician will be blinded to control/intervention group allocation. Descriptive statistics will be presented for demographic, baseline characteristics and endpoint data. Number and percentage will be presented for categorical data. Mean and standard deviation will be presented for normally distributed continuous data while median inter-quartile range will be presented for non-normally distributed continuous data.

The number of participants consented, randomised, and completing the study will be described in a study CONSORT diagram.

Data analysis will be performed on an Intention to Treat basis. Data will be analysed with the statistical software Stata version 17.0. All statistical tests will be 2-sided and significance set at $p < 0.05$.

For peer review only - http://bmjopen.bmj.com/site/about/guidelines.xhtml

Primary endpoint analysis

For each participant, diagnostic performance will be calculated as percentage of the 50 spirometry procedures where the final diagnosis was correctly identified. In each randomised group, the diagnostic performance will be summarised as the mean of the participants' diagnostic performance. A 2-sample unpaired t test will be used to assess the mean difference in correct response between the 2 randomised groups. The assumptions for the 2-sample t test will be tested and where the assumption for equal variances fails then the Welch t test will be used.

Randomisation will be stratified according to job role (General Practitioner: Yes or No) and Accreditation from Association for Respiratory Technology and Physiology (Performance Only, Interpretation Only, Performance and Interpretation, No accreditation), therefore an analysis of covariance will be used to compare the intervention and control groups by adjusting for the main effects of the stratified variables. To internally validate the estimate of the difference between the 2 groups nonparametric bootstrap estimation of difference will be done by taking 100 from the analysis of covariance model.

Secondary endpoint analysis

For continuous variables, the secondary endpoints will be compared between the control and intervention groups using the same approach as the primary endpoint.

The diagnostic prediction performance of the AI software alone compared with the reference diagnosis by the pulmonologists will also be reported.

Sub-group analysis

Subgroup analysis will be performed on the 20 spirometry traces where the confirmed diagnosis (reference standard) was COPD, as spirometry in primary care is most relevant to COPD diagnosis.

Missing data

Only participants who complete at least 70% of the spirometry cases will be included in the primary analysis. We will compare the baseline characteristics and demographics of those who completed < 70% of questions with those who completed ≥ 70% of questions to identify any systematic differences between completers and non-completers.

For peer review only

For peer review only - http://bmjopen.bmj.com/site/about/guidelines.xhtml

Data Collection

Data will be collected via the online platform Qualtrics which is general data protection regulations (GDPR) compliant. Further information is provided at https://www.qualtrics.com/uk/platform/gdpr/.

A user license has been obtained by Royal Brompton and Harefield Hospital to use this platform for research purposes. There will be one secure user log in for one member of the study team who will be unblinded. All other members of the research team will be blinded.

Data Management

All data will be handled in accordance with the Data Protection Act (2018), NHS Caldecott Principles, The UK Policy Framework for Health and Social Care Research, and the condition of the REC approval. The online platform (Qualtrics) will be used to collect data responses from each participant reviewing the spirometry traces. Qualtrics itself will act as a database and data will also be downloaded to an excel file/csv file/database) for back up and analysis. Database access will be strictly restricted through user-specific passwords to the authorised research team members. All returned data (Microsoft Forms and Excel spreadsheets) will be held on the H drive (Harefield Hospital) by the clinical team (CI and study team) and will be password protected and only accessible by the direct study team.

No participant identifiable data, beyond basic demographics, will be transferred and all participants will have a participant identification number. Data will be downloaded typically within one week of data collection on Qualtrics by members of the research team. The research team will undertake appropriate reviews of the entered data where appropriate for the purpose of data cleaning and will request amendments as required.

At the end of the study, the CI or their assigned delegate will review all the data for each participant to verify that all the data are complete and correct. At this point, all data can be formally locked for analysis.

Patient and Public Involvement

Patient representatives are included in the trial management group and will attend the quarterly meetings across the duration of the trial. The Deputy Head of Research and Innovation for Asthma

For peer review only

+ Lung UK (the largest UK Lung Health charity) is also part of the trial management group. Our research team includes two costed co-applicants who will co-lead the PPI elements of the project.

PPI co-applicant responsibilities include: setting and refining overall PPI strategy as the project progresses, reporting on PPI activities to the research management group, evaluation, monitoring and reporting of PPI e.g. using a PPI impact log , communicating with our PPI collaborators (Asthma+ Lung UK) as well end-users (GP federations / clinical commissioning groups) on the project status, synthesising results and conclusions of PPI activities and disseminating feedback, writing up PPI sections with public contributors for ethics applications, patient-facing documents and project reports.

Safety Reporting

The procedure proposed for this efficacy study will not affect the usual standard of care for participants or patients. The datasets will comprise spirometry records previously collected as part of clinical spirometry pathways in primary care. As such, this retrospective analysis, which will not involve delivery of an intervention nor a change in patient's usual clinical care, is unlikely to produce direct risk for participants.

The main study risks involve patient confidentiality, information governance and avoiding bias. To address this, all data analysed by parties outside the direct clinical team will be de-identified. Spirometry records will be anonymised and provided to clinicians on an online platform (Qualtrics) compliant with GDPR.

The research team (except the platform administrator) and trial statistician will be blinded to participants' group allocation until the completion of data analysis for the primary and secondary outcomes.

Protocol amendments

Any changes to the study protocol outlined in this paper will be approved by Health Research Authority (HRA) and study Sponsor Royal Brompton and Harefield Hospitals (RBHH), Guy's and St Thomas' NHS Foundation Trust (GSTFT). Two non-substantial amendments have been submitted and approved by HRA for this study: 1. Addition of a link to the study platform in the participant information; 2. An increase in target recruitment number (detailed in Methods section).

For peer review only

Ethics and Dissemination

Ethical Approval

This study has been reviewed and given favourable opinion by Health Research Authority Wales (Reference: 22/HRA/5023) and registered on Clinical trials.gov (Reference: NCT05933694).

Dissemination

To maximise the audience for dissemination of our findings, results from the study will be disseminated by presentations at relevant scientific meetings and conferences as well as by high impact peer-reviewed publications. We will also disseminate via presentations and newsletters to participants and key stakeholders, including Asthma + Lung UK who have been collaborators on this grant. The results will be shared with local and national primary and secondary care partners and networks, including British Thoracic Society, Primary Care Respiratory Society and International Primary Care Respiratory Group. A summary report will be shared on the Royal Brompton and Harefield website research pages.

Discussion

Spirometry is a key investigation for supporting diagnosis and disease monitoring in chronic respiratory disease. There is a clear need to improve the access, quality and interpretation of spirometry in primary care and other non-respiratory community settings which have many historic challenges, further negatively impacted by the COVID pandemic (8). AI decision-support software has shown potential in improving quality assessment and interpretation of spirometry and this study will evaluate its effectiveness in interpretation of real-world spirometry results assessed by clinicians working in primary care in the UK.

This study is the first randomised controlled efficacy study with an intervention designed to improve the performance of primary care clinicians in the interpretation of spirometry using AI and an important first step for clinicians to make informed choices about its use. It will directly test the effect of an AI-powered spirometry interpretation software in primary care using real-world traces reflective of the results reviewed in practice by clinicians.

We will advertise the trial and recruit nationally to maximise participation and variation in participant sample.

For peer review only - http://bmjopen.bmj.com/site/about/guidelines.xhtml

Funding statement: The study is being funded by National Institute for Health Research (NIHR) through an AI Award in Health and Care (Phase 3- Application: Grant number AI_AWARD02204). The study is also supported by the NIHR Leicester Biomedical Research Centre – Respiratory theme. ST is supported by the National Institute for Health Research ARC North Thames. RR is supported by the NIHR Oxford Biomedical Research Centre – Respiratory. RAE is supported by an NIHR Clinical Scientist fellowship. The views expressed in this publication are those of the authors and not necessarily those of the National Institute for Health Research or the Department of Health and Social Care.

Competing interests: WM is Honorary President of the Association for Respiratory Technology and Physiology (ARTP). MT is CEO of ArtiQ – a company that produces artificial intelligence enabled lung function support software. JM and KVO are employees of ArtiQ.

Authors' contributions: WDC-M and MT conceived the wider research plan. WDC-M, MT, RAE, RR, SSK, SJCT, ATP, NH, NS were co-applicants for the wider grant. All authors developed the design and plan for this study. WB, WDC-M, GD completed the statistical analysis plan which was approved by all authors and ATP provided statistical expertise for the wider grant. MT and ArtiQ team developed the intervention. NS, IJ and NSH led the patient and public involvement. APS and GE set up the study on the Qualtrics platform. GD, EEE and WDC-M drafted the initial manuscript. All authors (GD, EEE, GE, MT, RAE, RR, KPS, KVO, APS, DAS, ATP, JH, SJCT, NSH, SSK, IJ, NS, WB and WD-CM) contributed to the development of the study protocol and reviewed, commented and approved the manuscript.

Acknowledgements: We thank our patient and public involvement members for their contribution to the overall grant application and involvement in trail management meetings. We acknowledge Asthma + Lung UK for their contribution and support with this work.

Figure 1. Example spirometry case and questions for primary care clinicians

Figure 2. Study Schedule

References

1. Global burden of chronic respiratory diseases and risk factors, 1990-2019: an update from the Global Burden of Disease Study 2019. EClinicalMedicine. 2023;59:101936.

For peer review only - http://bmjopen.bmj.com/site/about/guidelines.xhtml

2. IHME G. Global Burden of Disease Study 2019 (GBD 2019) results. Institute for Health Metrics and Evaluation Seattle, United States; 2016.
3. Jones RC, Price D, Ryan D, Sims EJ, von Ziegenweidt J, Mascarenhas L, et al. Opportunities to diagnose chronic obstructive pulmonary disease in routine care in the UK: a retrospective study of a clinical cohort. Lancet Respir Med. 2014;2(4):267-76.
4. Collard HR, Loyd JE, King TE, Jr., Lancaster LH. Current diagnosis and management of idiopathic pulmonary fibrosis: a survey of academic physicians. Respir Med. 2007;101(9):2011-6.
5. Heffler E, Crimi C, Mancuso S, Campisi R, Puggioni F, Brussino L, et al. Misdiagnosis of asthma and COPD and underuse of spirometry in primary care unselected patients. Respir Med. 2018;142:48-52.
6. NICE. Chronic obstructive pulmonary disease in over 16s: diagnosis and management 2018 [Available from: https://www.nice.org.uk/guidance/ng115.
7. NICE. Asthma: diagnosis, monitoring and chronic asthma management. 2017 [Available from: https://www.nice.org.uk/guidance/ng80.
8. Doe G, Taylor SJ, Topalovic M, Russell R, Evans RA, Maes J, et al. Spirometry services in England post-pandemic and the potential role of AI support software: a qualitative study of challenges and opportunities. British Journal of General Practice. 2023;73(737):e915-e23.
9. van de Hei SJ, Flokstra-de Blok BMJ, Baretta HJ, Doornewaard NE, van der Molen T, Patberg KW, et al. Quality of spirometry and related diagnosis in primary care with a focus on clinical use. NPJ Prim Care Respir Med. 2020;30(1):22.
10. Hegewald MJ, Gallo HM, Wilson EL. Accuracy and Quality of Spirometry in Primary Care Offices. Ann Am Thorac Soc. 2016;13(12):2119-24.
11. Societies FoIR. The global impact of respiratory disease. Third Edition. European Respiratory Society. 2021 2021.
12. Das N, Verstraete K, Stanojevic S, Topalovic M, Aerts J-M, Janssens W. Deep-learning algorithm helps to standardise ATS/ERS spirometric acceptability and usability criteria. European Respiratory Journal. 2020;56(6):2000603.
13. Topalovic M, Das N, Burgel PR, Daenen M, Derom E, Haenebalcke C, et al. Artificial intelligence outperforms pulmonologists in the interpretation of pulmonary function tests. Eur Respir J. 2019;53(4).
14. Desbordes P, Topole E, Biondaro S, Montagna I, Corre S, Topalovic M. AI Over-reading Based on ATS/ERS 2019 Criteria Is a Reliable Option for Instant Spirometry Quality Control in Clinical Trials. B70 INHALE THE FUTURE: NEW FINDINGS IN PULMONARY FUNCTION. p. A4064-A.
15. Graham BL, Steenbruggen I, Miller MR, Barjaktarevic IZ, Cooper BG, Hall GL, et al. Standardization of Spirometry 2019 Update. An Official American Thoracic Society and European Respiratory Society Technical Statement. American Journal of Respiratory and Critical Care Medicine. 2019;200(8):e70-e88.

For peer review only - http://bmjopen.bmj.com/site/about/guidelines.xhtml

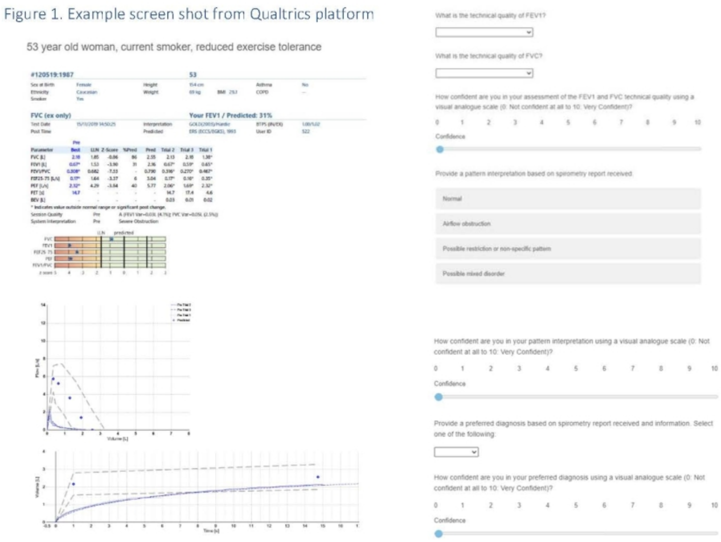

Figure 1. Example spirometry case and questions for primary care clinicians

210x297mm (200 x 200 DPI)

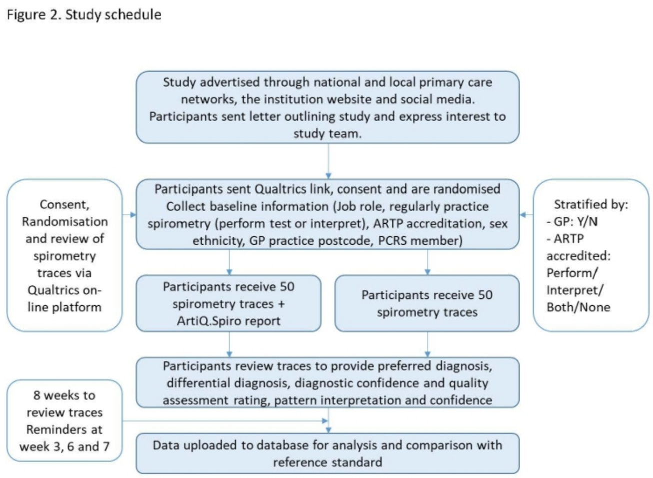

Figure 2. Study Schedule

210x297mm (210 x 210 DPI)

Supplement_Comparing performance of primary care clinicians in the interpretation of SPIROmetry with or without Artificial Intelligence Decision support software (SPIRO-AID): a protocol for a randomised controlled trial.

Supplement

Clinician letter (participant information)

Royal Brompton and Harefield

NHS Guy's and St Thomas' NHS Foundation Trust

IRAS ID: 323361

Invitation to participate in research study: A randomized controlled trial comparing diagnostic performance of primary care clinicians in the interpretation of **SPIRO**metry with or without **A**rtificial **I**ntelligence **D**ecision support software (SPIRO-AID)

Dear Clinician,

We would like to invite you to take part in our research study. This study aims to evaluate whether an artificial intelligence decision support software (ArtiQ. Spiro) improves the diagnostic accuracy of spirometry interpreted by primary care clinicians, as measured by Clinician Diagnostic Accuracy (*vs* Reference Standard). You are being invited to take part because you are a clinician working in primary care who refers patients for spirometry and/or receives spirometry results for review.

The study is being conducted by Harefield Hospital, part of Guy's and St Thomas's NHS Foundation Trust (GSTFT), in partnership with ArtiQ, a company specialising in respiratory medicine and Artificial Intelligence (AI). The Chief Investigator (CI) is Prof William Man, Professor and Honorary Consultant Respiratory Physician. The study is being funded by National Institute for Health Research (NIHR) through an AI Award in Health and Care (Phase 3- Application: Grant number AI_AWARD02204). The lead organisation for the wider research project is Guy's and St Thomas's NHS Foundation Trust. The study is also supported by the NIHR Leicester Biomedical Research Centre - Respiratory.

Please take time to read the following information carefully. Please discuss it with others, including your colleagues if you wish. Please ask us if there is anything that is not clear.

Purpose of the Study: The NHS Long Term Plan (LTP) has identified quality assured spirometry as a clinical priority through investment in training more community staff to perform and interpret spirometry more accurately. However, this process takes time and disagreement over spirometry interpretation can still occur between trained staff. The purpose of this study is to evaluate whether an AI decision support software improves the diagnostic accuracy and quality assessment of spirometry by primary care clinicians.

Taking part: If you are happy to take part in this research please click here where you will be provided with a link to an on-line platform (Qualtrics) to assess your eligibility in the study and to complete a Consent form. You will then be asked about your job role and any previous experience with spirometry, which will help the study team to randomise participants into each arm of the study. Some participants will be randomised to receive

SPIRO-AID Clinician Information Letter
Version 1.2, 7 March 2023
Page 1 of 3

For peer review only - http://bmjopen.bmj.com/site/about/guidelines.xhtml

Supplement_Comparing performance of primary care clinicians in the interpretation of SPIROmetry with or without Artificial Intelligence Decision support software (SPIRO-AID): a protocol for a randomised controlled trial.

50 spirometry traces for review in the usual format that they would be received. Some participants will be randomised to receive 50 spirometry traces plus an AI report (ArtiQ.Spiro). The 50 spirometry traces will be from a de-identified retrospective dataset. We anticipate that reviewing the traces will take approximately one hour of your time.

Expenses and payments: Participants will be offered reimbursement (£150) for their time. Reimbursement details are provided via the Qualtrics study platform after you have completed review of the 50 traces.

Risks and benefits of taking part: The procedure proposed for this study does not affect the usual standard of care for participants. The datasets will comprise spirometry data previously collected as part of clinical spirometry pathways in primary care. As such, this retrospective data analysis, which does not involve delivery of an intervention nor a change in patient's usual clinical care, is unlikely to produce direct risk for participants.

This study will help us to understand if the addition of AI improves diagnostic accuracy and quality assessment of spirometry, informing design of spirometry pathways for the future.

Study period: The study period will be six months. When this period is over, the results will be analysed and written up for dissemination. The results will be presented at scientific meetings and will be published in medical journals. A dissemination event may be arranged and we will invite participants to attend.

Confidentiality: All information which is collected about you during the course of the research will be kept strictly confidential. Royal Brompton and Harefield Hospitals, part of Guy's and St Thomas NHS Foundation Trust is the sponsor for this study. The sponsor will act as the data controller. This means that it is responsible for looking after your information and using it properly. A web based electronic platform (Qualtrics) will be used to collect data responses from each participant reviewing the spirometry traces.

How will we use information about you?

We will need to use information from you for this research project. This information will include your name and contact details. People will use this information to do the research or to check your records to make sure that the research is being done properly.

People who do not need to know who you are will not be able to see your name or contact details. Your data will have a code number instead. We will keep all information about you safe and secure. Database access will be strictly restricted through user-specific passwords to the authorised research team members. At the end of the study, the CI or their assigned delegate will review all the data for each participant to verify that all the data are complete and correct. At this point, all data can be formally locked for analysis.

For peer review only - http://bmjopen.bmj.com/site/about/guidelines.xhtml

Supplement_Comparing performance of primary care clinicians in the interpretation of SPIROmetry with or without Artificial Intelligence Decision support software (SPIRO-AID): a protocol for a randomised controlled trial.

Once we have finished the study, we will keep some of the data so we can check the results. We will write our reports in a way that no-one can work out that you took part in the study.

What are your choices about how your information is used?
You can stop being part of the study at any time, without giving a reason, but we will keep information about you that we already have.
We need to manage your records in specific ways for the research to be reliable. This means that we won't be able to let you see or change the data we hold about you.

Where can you find out more about how your information is used?
You can find out more about how we use your information

- at www.hra.nhs.uk/information-about-patients/
- by asking one of the research team
- by sending an email to ig@rbht.nhs.uk, or
- by ringing us on 0207 352 8121 ext. 2610.

Additional information on the use of patient data in research in line with the General Data Protection Regulation (GDPR) is also provided here.

If you have a concern about any aspect of this study you should ask to speak with the researchers who will do their best to answer your questions [Insert local contact number]. If you remain unhappy and wish to complain formally, you can do this. Details can be obtained from the Royal Brompton and Harefield Hospitals (RBHH) Research Office *via* Email: (Insert)

Further Information and Contact Details:
If you require any further information, please do not hesitate to contact us on the details below.

Study email: Insert

Royal Brompton and Harefield Hospital Research Team
Contact [Insert TBC]

Gillian Doe (Research Programme Manager)
Respiratory BRC, Leicester **Tel:** 0116 258 3370 **Email:** ged6@leicester.ac.uk

Yours faithfully,

[Insert signature]
Professor Will Man (CI)

For peer review only - http://bmjopen.bmj.com/site/about/guidelines.xhtml

Supplement_Comparing performance of primary care clinicians in the interpretation of SPIROmetry with or without Artificial Intelligence Decision support software (SPIRO-AID): a protocol for a randomised controlled trial.

Consent form on Study platform

Q15

Please initial each box if you are in agreement

	Initials
I confirm that I have read and understand the information in the Clinician invitation letter dated 07/03/2023 (Version 1.2) for the above study and have had the opportunity to consider the information, ask questions and have these answered satisfactorily.	
I understand that my participation is voluntary and that I am free to withdraw at any time, without giving any reason, without medical care or legal rights being affected.	
I understand that data collected during the study may be looked at by responsible individuals from the study Sponsor or from regulatory authorities where it is relevant to my taking part in research. I give permission to these individuals to have access this data.	
I agree with the publication of the results of this study in a medical journal (all data will be published anonymously).	
I agree to take part in the above study.	

Q16

Name of Participant

Q17

Date

${date://CurrentDate/FL}

Q21

Type your full name below in lieu of a signature

For peer review only - http://bmjopen.bmj.com/site/about/guidelines.xhtml

Supplement_Comparing performance of primary care clinicians in the interpretation of SPIROmetry with or without Artificial Intelligence Decision support software (SPIRO-AID): a protocol for a randomised controlled trial.

Example AI-decision support software report

ARTIQ

Session ID: 120519_1987
Analysed on: 2023-03-23 14:50:25 UTC±00:00

Session Evaluation

FEV1 (Grade A) Acceptable
FVC (Grade B) Acceptable

Best Trial Results
Best Trial: 1
FEV1: 0.7 L
FVC: 2.19 L

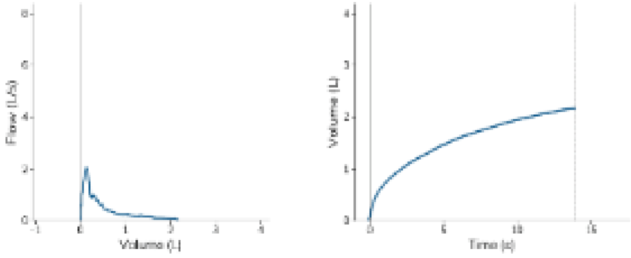

Trial Overview

	FEV1		FVC	
	Acceptable	Usable	Acceptable	Usable
Trial 1	Yes	-	Yes	-
Trial 2	Yes	-	Yes	-
Trial 3	Yes	-	No	Yes

Trial Evaluation Details

TRIAL 1
FEV1 Acceptable
FVC Acceptable

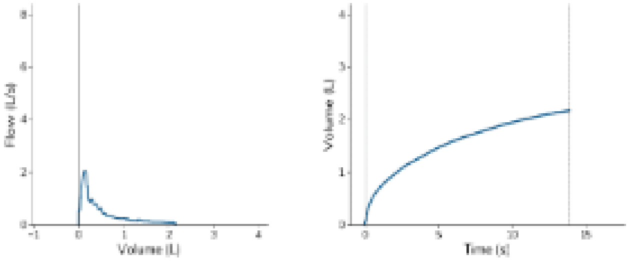

BEV Criteria: True
EOFE Criteria: True
Plateau Criteria: False
(39.35 mL change in last second)
tFE > 15s: False
FVC Repeatable: True
(30.34 mL lower than max acceptable FVC)
FIVC-FVC Criteria: None

No artefact on MEFVC

Warnings
No second inspiration

Supplement_Comparing performance of primary care clinicians in the interpretation of SPIROmetry with or without Artificial Intelligence Decision support software (SPIRO-AID): a protocol for a randomised controlled trial.

TRIAL 2

FEV1 Acceptable

FVC Acceptable

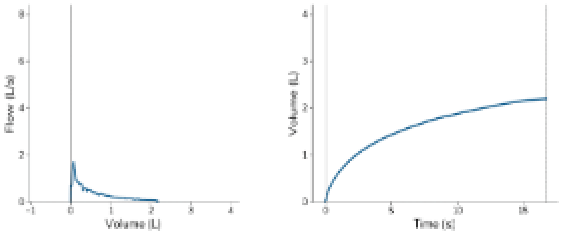

BEV Criteria: True

EOFE Criteria: True

Plateau Criteria: True

(19.9 mL change in last second)

tFE > 15s: True

FVC Repeatable: True

(0.0 mL lower than max acceptable FVC)

FIVC-FVC Criteria: None

No artefact on MEFVC

Warnings

No second inspiration

TRIAL 3

FEV1 Acceptable

FVC Usable (Failure of EOFE criteria)

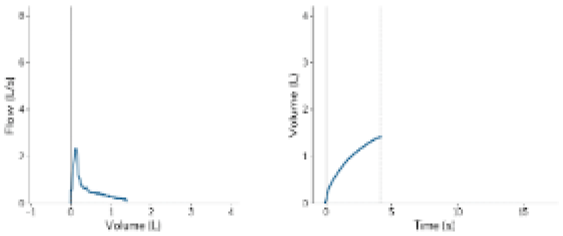

BEV Criteria: True

EOFE Criteria: False

Plateau Criteria: False

(143.41 mL change in last second)

tFE > 15s: False

FVC Repeatable: False

(780.41 mL lower than max acceptable FVC)

FIVC-FVC Criteria: None

No artefact on MEFVC

Warnings

No second inspiration

*Note no second inspiration present on these curves as this is a retrospective dataset where second inspiration was not captured

For peer review only - http://bmjopen.bmj.com/site/about/guidelines.xhtml

Supplement_Comparing performance of primary care clinicians in the interpretation of SPIROmetry with or without Artificial Intelligence Decision support software (SPIRO-AID): a protocol for a randomised controlled trial.

ARTIQ

Analyzed: 2023-04-18 23:33 UTC±00:00

Report ID: _120519_1987

Age: 53 **Gender:** Female **Current Smoker:** Yes **Pack-Years:** N/A

Interpretation of lung function tests

Spirometry demonstrates an obstructive pattern, but a restrictive component is not excluded.

Disease probability

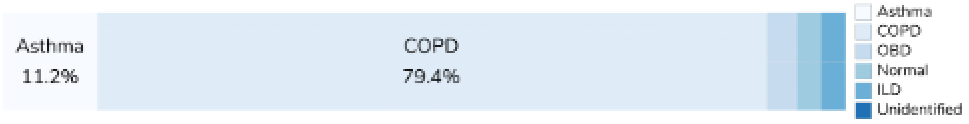

Conclusions and suggestions

Highest disease probability based on lung function: **COPD**

Consider referring to specialist and/or further testing as appropriate:
Careful auscultation on all lung areas for presence of rhonchi. Repeat spirometry with bronchodilator test. Perform screening laboratory (including eosinophilia). Perform X-ray of the thorax. Consider bronchodilator therapy.

Warnings

Probability of disease presence may not be accurate due to missing correct information of pack-years.

Legend

COPD	Chronic Obstructive Pulmonary Disease
OBD	Other Obstructive Diseases (including: cystic fibrosis, bronchiectasis, bronchiolitis)
Normal	Normal lung function
ILD	Interstitial lung disease (including: idiopathic pulmonary fibrosis, nonspecific interstitial pneumonitis, sarcoidosis)
Unidentified	Unidentified (including: neuromuscular disease, pulmonary vascular disease, thoracic deformity, pleural disease)

This report is approved for clinical use in the EU.
Automatically generated by ArtiQ.PFT VERSION NOT SET • Manufactured by ArtiQ NV • Leuven, Belgium

info@ArtiQ.eu
www.ArtiQ.eu

STANDARD PROTOCOL ITEMS: RECOMMENDATIONS FOR INTERVENTIONAL TRIALS

SPIRIT 2013 Checklist: Recommended items to address in a clinical trial protocol and related documents*

Section/item	ItemNo	Description	Page no.
**Administrative information**			
Title	1	Descriptive title identifying the study design, population, interventions, and, if applicable, trial acronym	1
Trial registration	2a	Trial identifier and registry name. If not yet registered, name of intended registry	2<br>Clinical trials.gov (Reference: NCT05933694).
	2b	All items from the World Health Organization Trial Registration Data Set	
Protocol version	3	Date and version identifier	Version 1 for Publication
Funding	4	Sources and types of financial, material, and other support	18<br>Funded by National Institute for Health Research (NIHR) through an AI Award in Health and Care (Phase 3-Application: Grant number AI_AWARD02204
Roles and responsibilities	5a	Names, affiliations, and roles of protocol contributors	1
	5b	Name and contact information for the trial sponsor	4<br>Royal Brompton and Harefield Hospitals (RBHH), Guy's and St Thomas' NHS Foundation Trust (GSTFT)

For peer review only

	5c	Role of study sponsor and funders, if any, in study design; collection, management, analysis, and interpretation of data; writing of the report; and the decision to submit the report for publication, including whether they will have ultimate authority over any of these activities	18
	5d	Composition, roles, and responsibilities of the coordinating centre, steering committee, endpoint adjudication committee, data management team, and other individuals or groups overseeing the trial, if applicable (see Item 21a for data monitoring committee)	18
**Introduction**			
Background and rationale	6a	Description of research question and justification for undertaking the trial, including summary of relevant studies (published and unpublished) examining benefits and harms for each intervention	3 and 4
	6b	Explanation for choice of comparators	
Objectives	7	Specific objectives or hypotheses	4
Trial design	8	Description of trial design including type of trial (eg, parallel group, crossover, factorial, single group), allocation ratio, and framework (eg, superiority, equivalence, noninferiority, exploratory)	4
**Methods: Participants, interventions, and outcomes**			

For peer review only

Study setting	9	Description of study settings (eg, community clinic, academic hospital) and list of countries where data will be collected. Reference to where list of study sites can be obtained	6
Eligibility criteria	10	Inclusion and exclusion criteria for participants. If applicable, eligibility criteria for study centres and individuals who will perform the interventions (eg, surgeons, psychotherapists)	5
Interventions	11a	Interventions for each group with sufficient detail to allow replication, including how and when they will be administered	10 and 11
	11b	Criteria for discontinuing or modifying allocated interventions for a given trial participant (eg, drug dose change in response to harms, participant request, or improving/worsening disease)	
	11c	Strategies to improve adherence to intervention protocols, and any procedures for monitoring adherence (eg, drug tablet return, laboratory tests)	
	11d	Relevant concomitant care and interventions that are permitted or prohibited during the trial	n/a
Outcomes	12	Primary, secondary, and other outcomes, including the specific measurement variable (eg, systolic blood pressure), analysis metric (eg, change from baseline, final value, time to event), method of aggregation (eg, median, proportion), and time point for each outcome. Explanation of the clinical relevance of chosen efficacy and harm outcomes is strongly recommended	12,13

Participant timeline	13	Time schedule of enrolment, interventions (including any run-ins and washouts), assessments, and visits for participants. A schematic diagram is highly recommended (see Figure)	10<br>Figure 2 – study schedule
Sample size	14	Estimated number of participants needed to achieve study objectives and how it was determined, including clinical and statistical assumptions supporting any sample size calculations	13 and 14
Recruitment	15	Strategies for achieving adequate participant enrolment to reach target sample size	14
**Methods: Assignment of interventions (for controlled trials)**			
Allocation:			
Sequence generation	16a	Method of generating the allocation sequence (eg, computer-generated random numbers), and list of any factors for stratification. To reduce predictability of a random sequence, details of any planned restriction (eg, blocking) should be provided in a separate document that is unavailable to those who enrol participants or assign interventions	5
Allocation concealment mechanism	16b	Mechanism of implementing the allocation sequence (eg, central telephone; sequentially numbered, opaque, sealed envelopes), describing any steps to conceal the sequence until interventions are assigned	
Implementation	16c	Who will generate the allocation sequence, who will enrol participants, and who will assign participants to interventions	5 and 6

For peer review only - http://bmjopen.bmj.com/site/about/guidelines.xhtml

Blinding (masking)	17a	Who will be blinded after assignment to interventions (eg, trial participants, care providers, outcome assessors, data analysts), and how	6, 12, 14 and 15
	17b	If blinded, circumstances under which unblinding is permissible, and procedure for revealing a participant's allocated intervention during the trial	
**Methods: Data collection, management, and analysis**			
Data collection methods	18a	Plans for assessment and collection of outcome, baseline, and other trial data, including any related processes to promote data quality (eg, duplicate measurements, training of assessors) and a description of study instruments (eg, questionnaires, laboratory tests) along with their reliability and validity, if known. Reference to where data collection forms can be found, if not in the protocol	15 and 16
	18b	Plans to promote participant retention and complete follow-up, including list of any outcome data to be collected for participants who discontinue or deviate from intervention protocols	9 and 10<br>Figure 2
Data management	19	Plans for data entry, coding, security, and storage, including any related processes to promote data quality (eg, double data entry; range checks for data values). Reference to where details of data management procedures can be found, if not in the protocol	15 and 16

Statistical methods	20a	Statistical methods for analysing primary and secondary outcomes. Reference to where other details of the statistical analysis plan can be found, if not in the protocol	13 and 14
	20b	Methods for any additional analyses (eg, subgroup and adjusted analyses)	15
	20c	Definition of analysis population relating to protocol non-adherence (eg, as randomised analysis), and any statistical methods to handle missing data (eg, multiple imputation)	15
**Methods: Monitoring**			
Data monitoring	21a	Composition of data monitoring committee (DMC); summary of its role and reporting structure; statement of whether it is independent from the sponsor and competing interests; and reference to where further details about its charter can be found, if not in the protocol. Alternatively, an explanation of why a DMC is not needed	15 and 16 – data management.<br>No DMC<br>Data will be monitored, checked and cleaned by the study team.
	21b	Description of any interim analyses and stopping guidelines, including who will have access to these interim results and make the final decision to terminate the trial	15
Harms	22	Plans for collecting, assessing, reporting, and managing solicited and spontaneously reported adverse events and other unintended effects of trial interventions or trial conduct	16 and 17

Auditing	23	Frequency and procedures for auditing trial conduct, if any, and whether the process will be independent from investigators and the sponsor	The Sponsor operates a risk based monitoring and audit programme, to which this study will be subject.
**Ethics and dissemination**			
Research ethics approval	24	Plans for seeking research ethics committee/institutional review board (REC/IRB) approval	2<br>This study has been reviewed and given favourable opinion by Health Research Authority Wales (Reference: 22/HRA/5023).
Protocol amendments	25	Plans for communicating important protocol modifications (eg, changes to eligibility criteria, outcomes, analyses) to relevant parties (eg, investigators, REC/IRBs, trial participants, trial registries, journals, regulators)	17
Consent or assent	26a	Who will obtain informed consent or assent from potential trial participants or authorised surrogates, and how (see Item 32)	5 and 6
	26b	Additional consent provisions for collection and use of participant data and biological specimens in ancillary studies, if applicable	n/a
Confidentiality	27	How personal information about potential and enrolled participants will be collected, shared, and maintained in order to protect confidentiality before, during, and after the trial	Supplement<br>- Clinician letter (participant information sheet)
Declaration of interests	28	Financial and other competing interests for principal investigators for the overall trial and each study site	18

For peer review only - http://bmjopen.bmj.com/site/about/guidelines.xhtml

Access to data	29	Statement of who will have access to the final trial dataset, and disclosure of contractual agreements that limit such access for investigators	
Ancillary and post-trial care	30	Provisions, if any, for ancillary and post-trial care, and for compensation to those who suffer harm from trial participation	Participation is considered to be very low risk and does not involve patients.
Dissemination policy	31a	Plans for investigators and sponsor to communicate trial results to participants, healthcare professionals, the public, and other relevant groups (eg, via publication, reporting in results databases, or other data sharing arrangements), including any publication restrictions	17
	31b	Authorship eligibility guidelines and any intended use of professional writers	
	31c	Plans, if any, for granting public access to the full protocol, participant-level dataset, and statistical code	
**Appendices**			
Informed consent materials	32	Model consent form and other related documentation given to participants and authorised surrogates	Supplement
Biological specimens	33	Plans for collection, laboratory evaluation, and storage of biological specimens for genetic or molecular analysis in the current trial and for future use in ancillary studies, if applicable	n/a

*It is strongly recommended that this checklist be read in conjunction with the SPIRIT 2013 Explanation & Elaboration for important clarification on the items. Amendments to the protocol should be tracked and dated. The SPIRIT checklist is copyrighted by the SPIRIT Group under the Creative Commons "Attribution-NonCommercial-NoDerivs 3.0 Unported" license.

For peer review only
